# Analysis of Sequence Variability and Transcriptional Profile of *Cannabinoid synthase* Genes in *Cannabis sativa* L. Chemotypes with a Focus on *Cannabichromenic acid synthase*

**DOI:** 10.3390/plants10091857

**Published:** 2021-09-08

**Authors:** Flavia Fulvio, Roberta Paris, Massimo Montanari, Cinzia Citti, Vincenzo Cilento, Laura Bassolino, Anna Moschella, Ilaria Alberti, Nicola Pecchioni, Giuseppe Cannazza, Giuseppe Mandolino

**Affiliations:** 1 CREA—Research Centre for Cereal and Industrial Crops, Via di Corticella 133, 40128 Bologna, Italy; flavia.fulvio@crea.gov.it (F.F.); massimo.montanari@crea.gov.it (M.M.); vincenzo.cilento@edu.unito.it (V.C.); laura.bassolino@crea.gov.it (L.B.); anna.moschella@crea.gov.it (A.M.); giuseppe.mandolino@crea.gov.it (G.M.); 2 Department of Sciences of Agriculture, Food Natural Resources and Engineering, University of Foggia, Via Napoli 25, 71122 Foggia, Italy; 3 CNR NANOTEC—Institute of Nanotechnology, Via Monteroni, 73100 Lecce, Italy; cinzia.citti@unimore.it (C.C.); giuseppe.cannazza@unimore.it (G.C.); 4 Department of Life Science, University of Modena and Reggio Emilia, Via G. Campi 103, 41125 Modena, Italy; 5 CREA—Research Centre for Cereal and Industrial Crops, Via G. Amendola 82, 45100 Rovigo, Italy; ilaria.alberti@crea.gov.it; 6 CREA—Research Centre for Cereal and Industrial Crops, S.S. 673 Km 25,200, 71122 Foggia, Italy; nicola.pecchioni@crea.gov.it

**Keywords:** *cannabinoid synthase*, SNPs, *CBCAS*, RT-qPCR

## Abstract

*Cannabis sativa* L. has been long cultivated for its narcotic potential due to the accumulation of tetrahydrocannabinolic acid (THCA) in female inflorescences, but nowadays its production for fiber, seeds, edible oil and bioactive compounds has spread throughout the world. However, some hemp varieties still accumulate traces of residual THCA close to the 0.20% limit set by European Union, despite the functional gene encoding for THCA synthase (THCAS) is lacking. Even if some hypotheses have been produced, studies are often in disagreement especially on the role of the cannabichromenic acid synthase (CBCAS). In this work a set of European *Cannabis* genotypes, representative of all chemotypes, were investigated from a chemical and molecular point of view. Highly specific primer pairs were developed to allow an accurate distinction of different *cannabinoid synthases* genes. In addition to their use as markers to detect the presence of *CBCAS* at genomic level, they allowed the analysis of transcriptional profiles in *hemp* or *marijuana* plants. While the high level of transcription of *THCAS* and *cannabidiolic acid synthase* (*CBDAS*) clearly reflects the chemical phenotype of the plants, the low but stable transcriptional level of *CBCAS* in all genotypes suggests that these genes are active and might contribute to the final amount of cannabinoids.

## 1. Introduction

*Cannabis sativa* L. is an annual, dioecious plant, characterized by the production of cannabinoids, terpenophenolic metabolites of great pharmaceutical interest, mainly synthetized and secreted in glandular trichomes of pistillate inflorescences.

Cannabinoids are produced by condensation of a phenolic moiety (usually olivetolic acid) with a terpenic one (geranylgeraniol-diphosphate). This reaction synthesizes cannabigerolic acid (CBGA), from which the other main cannabinoids are formed via enzymatic conversions.

Enzymatic cannabinoid biosynthesis is catalyzed by a number of oxidocyclases, among which the most prominent are the tetrahydrocannabinolic acid-, cannabidiolic acid- and cannabichromenic acid-synthase, leading to the accumulation of tetrahydrocannabinolic acid (THCA), cannabidiolic acid (CBDA) and cannabichromenic acid (CBCA) respectively [1]. These enzymes are thought to be poorly or completely non-functional in CBGA-accumulating plants [2].

Based on both the amount (expressed as% weight/weight) of main cannabinoids and their ratio, the chemical phenotype (chemotype) of *Cannabis* plants can be classified from I to V [2].

*THCA synthase* (*THCAS*), *CBDA synthase* (*CBDAS*) and *CBCA synthase* (*CBCAS*) are members of the *Berberine Bridge Enzyme* (*BBE*)*-like* gene family, containing an N-terminal signal peptide and a Flavin Adenin Dinucleotide (FAD) binding domain [3,4]. 

The genetics of cannabinoid synthesis has been studied for several years and different genes encoding these enzymes are known, each made of a single exon, with THCAS and CBCAS sharing 92% identity at amino acid level and 84% and 83% identity compared to CBDAS, respectively [3,4,5,6,7]. While *THCAS* and *CBDAS* have been widely studied at genetic and molecular level, little information is available on *CBCAS* genes.

Originally, Kojoma et al. [8] obtained from hemp varieties gene sequences highly related to functional *THCAS*, putatively encoding for complete polypeptides, but differing for several single nucleotide polymorphisms (SNPs). They named these sequences fiber-type *THCAS* genes, under the assumption that they were not functional and unable to synthesize cannabinoids [8]. Since then, a number of so-called fiber-type *THCAS* sequences have been found in both hemp and high THCA varieties [9,10]. 

In 2019, Laverty et al. [6] demonstrated that a fiber-type *THCAS* gene coded for a 71 kDa CBCAS, capable of transforming the CBGA precursor into CBCA and that the accumulation of CBCA correlated with the transcriptional level of *CBCAS* in various *Cannabis* tissues, with the highest level observed in female floral tissue. 

More recently, a comprehensive clade-based classification of all cannabinoid oxidocyclases proposed the naming of all fiber-type *THCAS* as *CBCAS* [7] since they belonged to the same clade of the only functionally CBCAS characterized by Laverty et al. [6]. Based on these results today they are conventionally collectively referred to as *CBCAS* and, according to this definition, in the present paper we will refer to all fiber-type *THCAS* sequences with a complete open reading frame as *CBCAS*.

Despite the name, further verification of their functionality *in planta* is needed and many authors claim for a meta-analysis to be conducted to verify that hypothesis [7,11]. Indeed, even though the CBCAS enzyme was biochemically characterized over 20 years ago [12] data on CBCAS are still scattered and somehow in disagreement. For example, one of the main inconsistencies with current evidence lies in previous analyses, where CBCA accumulation was demonstrated to be prominent during the juvenile stages of plant development, declining with maturity and irrespective of the chemotype at flowering [13].

Both the physiology of this enzyme and its relationship with the more studied CBDAS and THCAS are poorly understood and need clarification, especially in view of the possible contribution to the final chemotype. 

Understanding whether *CBCAS* sequences are present in each *Cannabis* variety or breeding material and the quantification of their transcription and potential translation *in planta*, would shed light on this important topic.

Several chemotype-associated PCR-based markers have been developed [10,14,15,16] to detect the presence of functional *THCAS* sequences, allowing to unambiguously distinguish between drug-type and fiber type materials. None of these markers amplify the *CBCAS* sequences, despite their high similarity at nucleotide level and among them, the three-primer marker system *B1080/B1192* developed by Pacifico et al. [15], is fully associated with the chemotype, being able to discriminate chemotype I (THCA prevalent), II (both THCA and CBDA present in roughly similar amounts) and III (CBDA prevalent).

A number of complete and partially annotated genomic sequences have been published in the latest years [6,17,18,19,20] and information about the transcribed genes in different stages and tissues [21], as well as on putative regulators of secondary metabolites [22,23] is already known. 

Despite the behavior as codominant alleles at a single locus observed in segregation analyses [24], *THCAS* and *CBDAS* genes have been recently mapped to separate loci in tight linkage on the same chromosome [6,19]. Moreover, in these loci there are multiple copies of *THCAS*- and *CBDAS*-related sequences. A few functional genes are expressed and therefore contribute to the final chemotype, while others are pseudogenes or partially functional sequences [21,25,26]. 

Interestingly, many hemp inflorescences accumulate traces of residual THCA at concentrations close and sometimes above the limit of 0.20% of dry weight set by E.U., despite the lack of a functional *THCAS* gene in their genomes and years of breeding aimed at eliminating the accumulation of this cannabinoid. Some hypotheses have been proposed: the first one is that the CBDAS could produce with very low efficiency THCA from CBGA, due to its high similarity with THCAS [27]; alternatively, sequences corresponding to putative *CBCAS* could be responsible for this residual production of THCA, small but apparently difficult to eliminate, since the substitutions at the protein level with functional THCAS are minimal and do not involve protein active sites or residues required for THCA synthesis [3,5]. Gaining a deeper knowledge of *CBCAS* sequences and of their transcription levels could help to better define the process behind the synthesis of THCA in hemp genotypes as an unexpected by-product and consequently guide future breeding strategies.

In the present work, several fiber- and drug-type genotypes were investigated for the presence of *CBCAS* sequences, assessing sequence variability and transcriptional levels. Markers were also developed to distinguish these sequences from the *THCAS* and *CBDAS* ones.

## 2. Results

### 2.1. Genotyping

Eleven hemp and two medical *Cannabis* genotypes (these last two currently used by pharmaceutical industry in Italy) were analyzed using the multiplex primer marker system *B1080/B1192* [15].

A single amplification band at 1192 bp was obtained for CINBOL (chemotype I, the only THCA-predominant variety), while for CINRO (chemotype II) both bands of expected size were amplified (Appendix A). For all hemp genotypes, the multiplex primer system amplified the 1080 band, with no distinction between chemotype III (Fibrante, Fibranova, Carmagnola, CS, Eletta Campana, Codimono, Carmaleonte and Futura 75), chemotype IV (Santhica 27 and Bernabeo) and Ermo (chemotype V), as already previously reported [15].

### 2.2. Quantification of Major Cannabinoids

The total amount of the main cannabinoids CBD, CBG and THC was measured by HPLC in female or monoecious hemp inflorescences harvested as reported in Table 1, about 4–5 weeks after full bloom, which would correspond, at least for day-length-sensitive genotypes, to the highest concentration of CBD or THC. Inflorescences of CINBOL and CINRO were collected earlier, to prevent an excessive accumulation of THC beyond the authorized limit for open field cultivation; therefore, total cannabinoid content was expected to be lower than at maturity (up to 18% in CINBOL and 15% in CINRO). 

The varieties with the highest cannabinoid content were CS and Carmagnola, with 5.49% and 5.32% CBD/d.w, respectively (Table 1). The other chemotype III genotypes (usually characterized by relatively high amounts of CBD) showed a CBD content ranging from 4.02% in Codimono to 1.92% in Carmaleonte. In Santhica 27, one of the lowest amounts of CBD (0.26%) was detected, followed by Ermo, which showed the lowest CBD content of all studied genotypes, as expected from its zero cannabinoids chemotype V. A high CBD content was found in CINRO, while CINBOL showed relatively very low amount of CBD in its female inflorescences.

Total CBG ranged between 0.10% in Carmaleonte to 2.69% *w/w* in Bernabeo; it was not detected in Ermo. 

All chemotype III genotypes had THC content below 0.20%, the legal limit for industrial hemp in E.U., while, as expected, the two medical varieties CINBOL and CINRO exceeded the limit up to 3.71% and 1.57%, respectively. Among hemp varieties, CS reached the highest residual THC content (0.17%), followed by Carmagnola and Eletta Campana. The lowest THC content and the highest CBG content was registered for Santhica 27 and Bernabeo (chemotype IV), while no trace of THC was detected in Ermo. CBC was under the limit of quantification for all but the two medical varieties, with a higher content in the CINRO inflorescences (0.02%). 

### 2.3. Diversity in CBCAS Sequences 

Specific primers were designed following a search for the “fiber-type *THCAS*” reference sequence, AB212830 [8], in the genome assemblies available on NCBI in 2019, listed in Supplementary Table S1.

Complete Open Reading Frames (ORFs), pseudogenes and fragments of a few hundred base pairs were found, but only the complete sequences, with at least 99% query cover and more than 99% identity were further considered. At least one *CBCAS* homolog putatively encoding for a complete protein was retrieved from each genome assembly, except for Chemdog 91 and LA Confidential.

The alignment of the complete ORFs to the AB212830 sequence revealed the presence of three conserved SNPs, in position 13, 18 and 1628 from the starting codon. The same SNPs were always confirmed aligning the ORFs derived from genome assemblies to the other available putative *CBCAS* genes deposited in public repositories. Based on this finding, a primer pair was designed (*CBCAS-cds* Fw and Rv, Table 2) including these three SNPs, which help to further differentiate these genes from *THCAS* and *CBDAS*. An alignment of one representative sequence for each genome against the reference sequence AB212830 is provided in Supplementary File S1, where these three polymorphisms are highlighted.

In order to investigate the *CBCAS* sequence variability within the set of 13 genotypes chosen in this work, PCR amplicons obtained with specific primers, covering the entire length of the coding sequence, were cloned and Sanger sequencing was performed on recombinant plasmids. All the putative *CBCAS* sequences were obtained from a pool of genomic DNA from 10 individual plants for each genotype; sequencing led to the identification of both pseudogenes and full-length coding sequences. A total of 77 *CBCAS* sequences were obtained, among which 30 unique sequences with a new and complete ORF were submitted to GenBank with ID numbers: MW429515- MW429540, MW429551, MW561073- MW561075.

**Figure 1:**
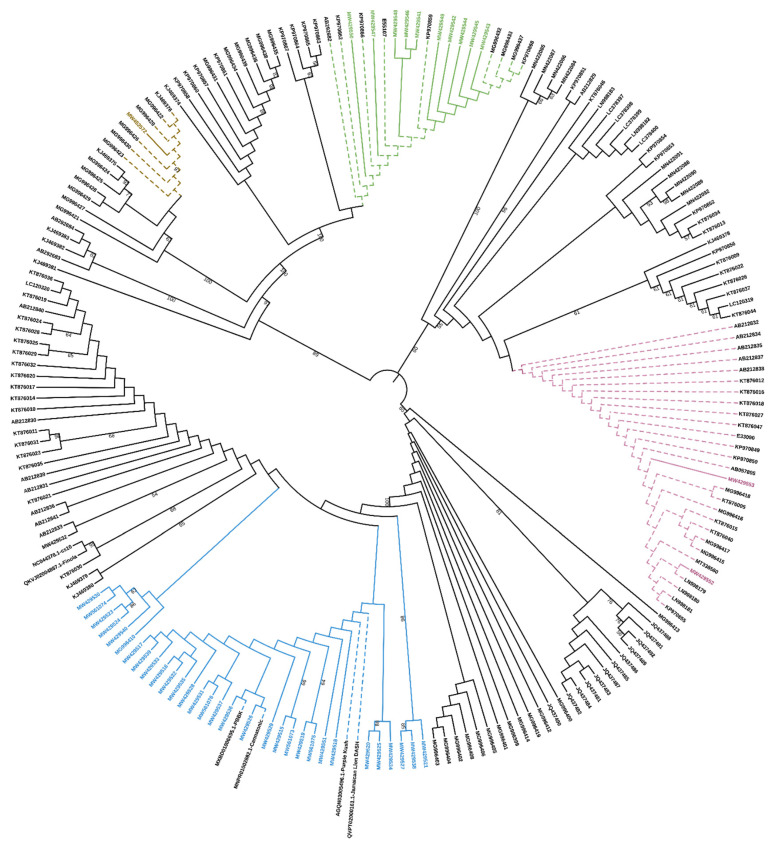
Phylogenetic tree analysis of 202 cannabinoid synthase genes from *C. sativa* at nucleotide level. The tree colors reflect the scheme presented by Van Velzen and Schranz in their Supplementary Figure S1. New *CBCAS* sequenced in the present work are in blue (clade A2), in pink new *THCAS* (clade A1) and in green new *CBDAS* sequences (clade, B, brown for clade B2-CBDAS). The optimal tree with the sum of branch length = 0.49420587 is shown. Branches corresponding to partitions reproduced in less than 50% bootstrap replicates are collapsed. The percentage of replicate trees in which the associated taxa clustered together in the bootstrap test (1000 replicates) are shown next to the branches [28]. The evolutionary distances were computed using the Maximum Composite Likelihood method [29] and are in the units of the number of base substitutions per site. Codon positions included were 1st + 2nd + 3rd + Noncoding. All ambiguous positions were removed for each sequence pair (pairwise deletion option). There were a total of 1906 positions in the final dataset.

To properly classify these sequences, they were aligned to the sequences contained in Supplementary File S1 of the work by van Velzen and Schranz [7] and a phylogenetic analysis based on the nucleotide sequences showed that they belong to the clade A2, comprising the only functionally characterized *CBCAS* and other putative *CBCAS* (Figure 1).

One to six *CBCAS* full cds were found in each *Cannabis* genotype analyzed in this work (Supplementary Table S2). The highest sequence variability was found in variety CS, with 6 different sequences, followed by Carmagnola, Carmaleonte and Fibrante, which have 5 different sequences each. 

Several recurrent sequences were present in more than one genotype, such as MW429551 (Carmaleonte, Fibrante, Santhica 27, Bernabeo, Ermo, CINRO and CINBOL), MW429517 (CS, Futura 75, Carmaleonte), MW429518 (Carmagnola and Fibranova) and MW429528 (CS and Fibrante), whereas the other sequences were identified only in one out of 13 genotypes under investigation.

When compared with the AB212830 reference sequence, the alignment of the 30 new sequences revealed SNPs in 42 different positions of the cds, 28 of them resulting in either amino acid transitions or transversions. Sequences differed by a variable number of SNPs, from three (MW429517, MW429522, MW429528, MW429529, MW429534, MW429551) up to eight (MW429523). 

The list of SNPs identified in the *CBCAS* sequences with the corresponding position and any amino acid substitutions is reported in Supplementary Table S3.

The similarity of the new cds with the AB212830 ranges from 99.51% (MW429520, MW429524) to 99.82% (MW429551), while the percentages of similarity with the *THCAS* (E33090) range from 95.72% (MW429523) to 96.02% (MW429534, MW429551).

### 2.4. Diversity in THCAS Sequences

Complete *THCAS* sequences were found only in the two medical *Cannabis* varieties, CINBOL and CINRO. All the eight sequences retrieved from CINRO (chemotype II) were identical to the reference functional *THCAS* sequence E33090. The variety CINBOL (chemotype I) showed higher variability, as three different sequences were found (E33090, MW429552, MW429553), out of seven sequenced complete cds. 

The possible presence of these sequences in databases was checked by BlastN against the standard NCBI non-redundant database, filtered by organism (*Cannabis sativa*, taxid: 3483) and no sequence with 100% identity was found. 

The two novel sequences found in CINBOL variety are respectively 99.94% and 99.88% identical to E33090. Only one SNP (A→986C) identified in MW429553 caused one amino acid change (N→328T) outside the three main domains of the putative translated protein. 

### 2.5. Diversity in CBDAS Sequences

Among 65 sequenced clones, 10 unique sequences putatively encoding for functional proteins were identified. From one (Carmagnola, Futura 75, Santhica 27, Bernabeo, CINRO) up to four (Carmaleonte, Ermo) different complete coding sequences have been found in each genotype (Supplementary Table S2).

No complete *CBDAS* cds was identified in CINBOL (chemotype I), in agreement with other studies [9,30,31].

Compared to the reference *CBDAS* gene (GenBank accession E55107), from one to four SNPs were found in 11 different positions. These SNPs differentiating each sequence are listed in Supplementary Table S4 and seven of them cause amino acid substitutions. All sequences share a common SNP respect to the reference sequence in position 105, where G was replaced by T, as already observed [25] This change is synonymous and therefore cannot affect the functionality of the resulting protein. 

Overall, the percentages of similarity between all identified sequences and the reference *CBDAS* sequence (E55107) are very high.

The most divergent sequence from E55107 is the *CBDAS* isolated from the chemotype V accession Ermo (MW429543) (99.76%), while the closest sequence is the MW429550 (99.94%), detected in 10 out of 13 genotypes. 

### 2.6. Transcriptional Analysis of Cannabinoid synthase Genes

The transcriptional level of the three classes of cannabinoid biosynthetic genes has been estimated by RT-qPCR in *Cannabis* inflorescences of all genotypes under study.

Given the high similarity of the target genes, the specificity of the designed primer pairs was accurately validated in three steps. Firstly, an in silico validation through blasting the primers to known reference sequences ensured that they perfectly matched exclusively sequences corresponding to the target genes. Only primers that generated perfect matches passed through the second validation step. This step was performed by PCR on different templates: genomic DNA, one plasmid containing the specific gene targeted by the primer pair (positive control) and one or more plasmids containing other non-target *cannabinoid synthase* genes (negative controls). Only the primer pairs that did not produce a band for the negative controls were brought to the next step (Appendix B). The same controls were then used in RT-qPCR analyses. In some cases, the specificity was gained after decreasing the primer concentration. Finally, amplification specificity was validated through melting curve analyses. Primer pairs showing a single high peak, indicating the absence of either non-target amplifications or primer dimers, were selected for the RT-qPCR assay; the optimal settings ensuring only specific target amplification are reported in Table 3. The melting curves for each specific amplicon are reported in Appendix B.

**Figure 2:**
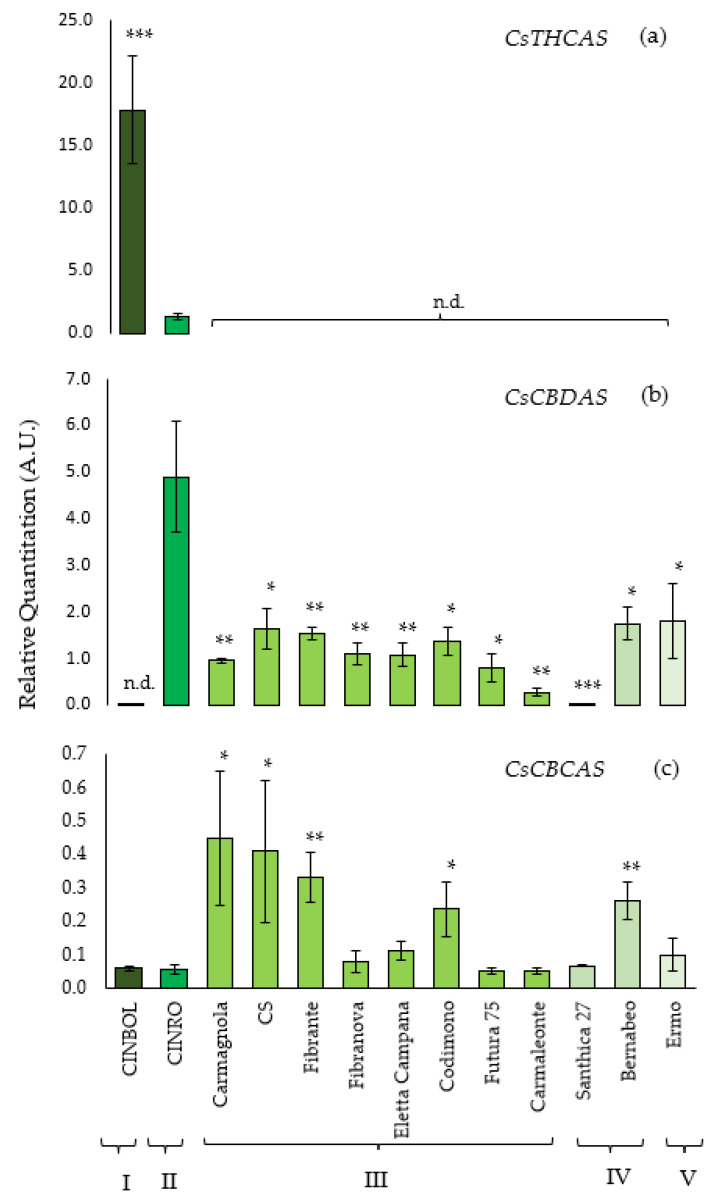
Relative Quantitation (R.Q.) of *CsTHCAS* (**a**), *CsCBDAS* (**b**), *CsCBCAS* (**c**) transcriptional levels in monoecious or female inflorescences. Y axis reports R.Q. expressed in Arbitrary Units (A.U.). Bars represent the standard errors of the mean of three biological replicates (*n* = 3). No detectable transcription levels are indicated as n.d. Below, for each genotype the corresponding chemotype is indicated (from I to V). Asterisks indicate Student’s t-test statistically significant differences: * *p* < 0.05; ** *p* < 0.01 and *** *p* < 0.001.

The transcriptional level of *THCA synthase* was analyzed in all 13 genotypes, but it could be detected and quantified only in CINBOL and CINRO varieties. The highest amount of *THCA synthase* transcripts was found in CINBOL (chemotype I, Figure 2a), where it was about 15 times higher than the transcription level detected in CINRO (chemotype II). On the contrary, *CBDA synthase* gene was transcribed in almost all samples. The highest transcription level was found for CINRO (R.Q. = 4.9, Figure 2b), while it was undetectable in CINBOL, where no functional gene encoding for a *CBDAS* was indeed found in this work. The *CBDAS* transcript levels varied among hemp varieties (R.Q. between 0.28 and 1.81), except for Santhica 27 where a very low transcript level was detected (R.Q. = 0.02).

The transcription level of the putative *CBCAS* genes has been investigated using the primers designed to avoid the amplification of non-target genes as *THCAS* and *CBDAS* (Table 3; Figure 2c) while amplifying all putative isoforms isolated in this work. In general, the *CBCAS* sequences were transcribed in all genotypes under examination, with a lower transcription level compared to *THCAS* and *CBDAS* genes. Similar levels were observed for Carmagnola, CS, Fibrante, Codimono and Bernabeo; lower ones were detected in Ermo, Santhica 27, Carmaleonte, Futura 75, Eletta Campana, Fibranova, CINRO and CINBOL. Sequencing of RT-qPCR products further confirmed that the expressed genes of CINBOL and CINRO corresponded to the *CBCAS* genes (data not shown).

In CINRO, the only variety where all three genes are expressed, *THCAS* transcription levels (R.Q.= 1.32) are about four times lower than *CBDAS* levels (R.Q. = 4.90) and 24 times higher than *CBCAS* (R.Q. = 0.05). In CINBOL the expression of *THCAS* (R.Q. = 17.80) is about 300× the one of *CBCAS* (R.Q. = 0.06). 

In general, in industrial hemp genotypes, where THCAS could not be detected, *CBDAS* transcript levels were higher than *CBCAS* ones (from 2× in Carmagnola up to 18× in Ermo). *CBCAS* was transcribed more than *CBDAS* only in Santhica 27 (Supplementary Table S5). 

To the best of our knowledge, this is the first report about the transcription of putative *CBCAS* genes related to the cannabinoid synthases and likely involved in CBCA synthesis and about a low but consistent presence of their mRNA in mature inflorescences in all tested samples, drug- but also hemp genotypes. 

## 3. Discussion

The current knowledge of *Cannabis sativa* has been shaped by a growing number of studies regarding its therapeutic potential and the wide applications of its main bioactive compounds, mostly cannabinoids, turning this plant from a criminalized drug to a multibillion-dollar business in only a few years [32]. Recently the United Nations Commission on Narcotic Drugs agreed on the removal of *Cannabis* from Schedule I of the Controlled Substances Act and this is thought to boost research into medical *Cannabis*, giving the plant a well-deserved attention and recognizing the therapeutic properties of its bioactive metabolites. 

As more studies on Eurasian landraces are required to unveil the genetic heterogeneity of the species [33], the present work focused on a set of Italian and French registered or patented *Cannabis* cultivars. The sequence variability and transcriptional levels of three *cannabinoid synthases* were investigated, focusing on *CBCAS*, reported for the first time in 2006 as fiber-type *THCAS* [8] and for many years neglected from the functional and genetic point of view. 

In this work, many complete and putatively functional *CBCAS* sequences were retrieved from both fiber- and drug-types genotypes, using a primer pair newly developed after in-silico analysis of different publicly available *Cannabis* assemblies.

Interestingly, a variable number of *CBCAS* sequences was obtained from the genotypes under study, ranging from one in Codimono, CINRO and CINBOL to up to six different sequences in the case of CS, some of them recurring (e.g., MW429551, MW429517, MW429518, MW429528) in more than one variety, while others specific to one genotype suggesting a wide distribution and variability among genotypes. 

Overall, these putative *CBCAS* sequences confirmed a great conservation of the SNPs differentiating this class of genes from the *THCAS* genes, as previously reported [8,9,10]. This diversification from *THCAS* and the high degree of conservation among genotypes would support the hypothesis that these genes could exert a specific role *in planta* [6].

A role in the biosynthesis of CBCA was demonstrated only for one sequence [6]. Whether (and if) the expression of these sequences correlates with the ability of the different varieties to produce CBCA in some stage of their development and consequently if they should all be considered as actually involved in CBCA biosynthesis *in planta*, is still to be determined. This is true not only for the sequences identified in this work, but also for all other putative *CBCAS* sequences released so far [7]. 

A recent phylogenetic analysis and subsequent classification of cannabinoid synthases has led to the definition of three different clades (A–C) [7]. The *CBCAS* retrieved in this work are included in the A2 clade, comprising full coding *CBCAS* genes and sequences defined as “incomplete”, “inactive” or “obscure” *THCAS*.

As expected, our *CBCAS* are in a separate branch compared to other Sanger sequenced cds, due to the presence of three SNPs at positions 13, 18 and 1628 bp, which deserve to be added to those previously identified as highly distinctive from *THCAS* [8,10].

Here, an assay was specifically developed to identify and study the transcription of *CBCAS*, allowing to detect the low expression of these genes in all the analyzed genotypes.

Weakly expressed *CBCAS* were found also in an RNA-seq experiment from female flower buds of nine *Cannabis* strains, where no detectable amounts of cannabichromene were found [34] In addition, Onofri et al., reported a small, yet detectable expression in pure-CBDA accessions [25].

According to Laverty et al. [6], the highest expression levels of *CBCAS* were found in female flowers and trichomes rather than in leaves. However, the primers used in Laverty’s work to analyze the *CBCAS* transcription by RT-qPCR are not specific and can actually target a *CBDAS*, as verified after BlastN vs. non redundant nucleotide database. On the other hand, de Meijer et al. [13] determined that CBCA accumulates predominantly in juvenile leaves and not in flowers. Taken together, these data made us sceptic about actuality and completeness of knowledge on *CBCAS*, that strongly encourages further analyses and verification of the function of these genes.

Grassa et al. [19] analyzed *cannabinoid synthase* genes expression by sequencing full length cDNA transcripts from high-CBD cultivars CBDRx (in leaves) and First Light (in flowers). No open reading frame for *CBCAS* was found, probably due to the low transcriptional level of the gene in the analyzed samples, compared to other *cannabinoid synthases*. 

Braich et al., using an RNA-seq approach, did not reveal *CBCAS* expression; however, this could be probably due to the misleading bioinformatics analyses of the short fragments deriving from paralogues and/or pseudogenes, all mapped to a same locus [21]. 

The analyses of transcriptional levels by using highly specific primers, as done in the present work, avoid the creation of misleading artifacts sometimes resulting by using short reads and coupled bioinformatics methods to analyze *Cannabis* transcriptomic massive outputs.

Different studies suggested the possibility that residual THCA in hemp plants is an apparently unavoidable by-product of CBDA synthesis by CBDAS; THCAS and CBDAS, in fact, have very similar kinetic characteristics (V_max_, K_M_, turnover) and this might explain the simultaneous formation of these two cannabinoids from the common precursor CBGA [27,28], though with different efficiencies. However, assuming this hypothesis was true, the issue about the existence of highly inbred lines accumulating practically only one single cannabinoid [24] would remain unexplained. 

An alternative explanation is that, given their ubiquity in *Cannabis* germplasm, *CBCAS* sequences likewise could be responsible for the low levels of THCA accumulation in hemp varieties at maturity, with occasional overcoming of the 0.20% d.w. THC threshold in some cultivations. The availability of specific markers, such as those presented in this work, could allow the counter-selection of these sequences during breeding, or their silencing by biotechnological strategies in a gene-specific and copy number-independent way, in order to definitively clarify the issue.

Comparing protein sequences of THCAS with CBCAS found in this work showed that there are no amino acid substitutions at residues necessary for the protein activity (from Arg110 to His114 and Cys176) or in residues whose substitution could affect its functionality, as demonstrated before [3,5]. Therefore, the possibility cannot be ruled out that the resulting CBCAS proteins, when translated, synthetize some THCA from CBGA precursor.

Based on our results, it appears that *CBCAS* is transcribed at low levels in all genotypes and that the THCA amount is also low in hemp varieties; it cannot be overlooked that varieties accumulating higher residual THC (Carmagnola, CS, Fibrante, Codimono) also share higher amounts of *CBCAS* transcripts (Table 1, Figure 2c).

Opposite to *CBCAS*, functional *THCAS* have been isolated in this work only from the medical varieties CINRO and CINBOL. In CINRO, only a *THCAS* sequence was retrieved from the genome, suggesting the presence of a single copy gene. Three different complete sequences of *THCAS* have been obtained from chemotype I CINBOL, suggesting the presence of at least two and up to three distinct copies of the gene. Both new sequences belong to the Subclade A1 from van Velzen and Schranz, with Type 1/1 (MW429552) and Type 1/2 (MW429553) differing by a single conservative substitution and therefore probably encoding for a functionally equivalent protein [25].

It has been suggested that reported variations in *THCAS* copy number had to be attributed to the use of promiscuous primers or to the unspecific inclusion of *CBCAS* sequences instead of only functional *THCAS* [7]. In the present study, the selective amplification and sequencing of cds from clones and their phylogenetic analysis represent stronger evidence of the presence of more than one copy of *THCAS* gene per single genotype (CINBOL).

Moreover, a Copy Number Variation (CNV) between CINBOL and CINRO was supported by preliminary RT-qPCR data, showing that the amplification plot from the genomic DNA of CINBOL crossed the threshold line earlier than those from CINRO (data not shown), as expected if CINBOL had more copies of the target gene. 

Whether or not this hypothetical CNV has a direct effect on the higher transcriptional level of *THCAS* and the higher amount of THCA in CINBOL in respect to CINRO is still to be determined. Other players like transcription factors or environmental conditions may be responsible for differences in transcriptional levels of these genes and cannabinoid accumulation [23]. 

Regarding the *CBDA synthases* class, a *CBDAS* gene has been sequenced (MW429549), with a SNP at position 1426, responsible for an amino acid substitution at position 476 of the enzyme (Pro → Ser). This substitution was already observed in Ermo and in another CBG-prevalent accession by Onofri et al. [25] and might result in a minimally functional CBDAS, unable to synthesize CBDA and leading to the accumulation of CBGA. This sequence probably accounts for chemotype IV when there are no other functional CBDAS, as in the case of Santhica 27 and Bernabeo. 

## 4. Materials and Methods

### 4.1. Plant Material and Samples Collection

Nine *Cannabis sativa* hemp varieties, two hemp selections and two medical varieties were used in this work. Among hemp, six were dioecious (Carmagnola, CS, Fibrante, Fibranova, Eletta Campana, Bernabeo) and five monoecious (Ermo, Santhica 27, Carmaleonte, Codimono and Futura 75). Medical varieties CINBOL and CINRO (CPVO registration numbers 50407 and 50406, respectively) were developed at CREA, cultivated in authorized indoor facilities and clonally propagated by cuttings.

For DNA isolation, seeds of the eleven hemp genotypes were sown in a peat-type TRAYSUBSTRATE (Klasmann-Deilmann Gmbh) and seedlings were grown in a plant growth chamber (Percival^®^AR-36LC8, Percival scientific, Inc. Perry, IA, USA), with a light/dark cycle of 18/6 h, a temperature of 24 °C and 60% relative humidity. About 14 days after sowing, young leaves were collected from 10 different seedlings of the same genotype, pooled and conserved at −80 °C until DNA extraction. 

For RNA isolation, the seeds of hemp genotypes were sown on the 19 April 2019 and cultivated in open field in CREA-CI experimental station of Rovigo (GPS coordinates: 45.078722/11.766035). Three biological replicates were collected from three different plants for each genotype, each consisting of three female or monoecious 10 cm apical flowers (comprising apical flower and floral leaves) sampled 123 days after sowing from the monoecious varieties and 151 days after sowing from the dioecious ones. 

**Figure 3:**
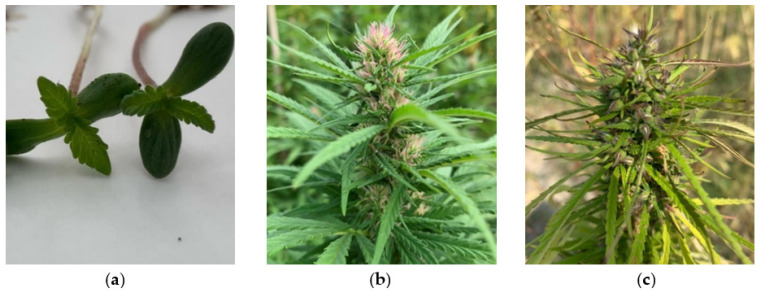
(**a**) *Cannabis* samples collected for DNA extraction, molecular cloning and sequencing. (**b**) CINRO female inflorescence 50 days after onset flowering. (**c**) Chemotype III dioecious genotype female inflorescence 151 days from sowing.

Samples (Figure 3) were immediately frozen in liquid nitrogen and stored at −80 °C.

Medical varieties with high THCA CINRO and CINBOL were also grown outdoor starting from cuttings from mother plants. For DNA extraction expanded leaves were collected from young, rooted cuttings; for RNA extraction, inflorescences were collected 50 days after the start of flowering, in the attempt to prevent an accumulation of THCA beyond the limit authorized for open field cultivation in Rovigo station. Samples were conserved as described above.

For chemical analysis, the same inflorescences were used after lyophilization.

### 4.2. LC-UV Analysis

For quantitative analysis of standard cannabinoids, namely CBDA, CBGA, THCA, CBD, CBG, Δ^9^-THC, Δ^8^-THC and CBC, samples (500 mg inflorescence of each variety, finely powdered) were prepared and analyzed by liquid chromatography coupled with UV detection (HPLC-UV) according to the protocol of the German Pharmacopoeia as previously reported [35,36]. Briefly, the extraction in analytical grade ethanol 96% (Carlo Erba, Milan, Italy) was carried out in three cycles with progressively decreasing volumes of solvent (20, 12.5 and 12.5 mL) under magnetic stirring for 15 min each. After collecting the liquid fraction in a volumetric flask, the extract was brought to 50 mL final volume with fresh ethanol. A 1 mL aliquot was filtered through a 0.45 µm syringe filter, diluted 1:10 with acetonitrile and injected into the chromatographic apparatus (5 µL). The chromatographic separation was performed on a Vanquish Core UHPLC system (Thermo Fisher Scientific, Waltham, MA, USA) equipped with a vacuum degasser, a binary pump, a thermostated autosampler at 4 °C, a thermostated column compartment set at 30 °C and a diode array detector. The analyses were carried out following the method reported in previous works [37,38,39] with slight modifications and acquired with the Chromeleon 7.3 Data System software (Thermo Fisher Scientific) following the UV signal registered at 228 nm. The column employed was a Poroshell 120 C18 column (Poroshell 120 EC-C18, 3.0 × 150 mm, 2.7 µm) (Agilent, Milan, Italy) and the mobile phase consisted of water (solvent A) and acetonitrile (solvent B) both with 0.1% formic acid. A linear gradient from 5 to 95% B was set over 20 min, then the column was washed with 95% B for 5 min and reconstituted with the initial mobile phase (5% B) for other 5 min bringing the total run time to 30 min.

Calibration curves were individually prepared in acetonitrile for each cannabinoid standard from 1 mg/mL stock solutions (Cerilliant, Sigma Aldrich, Milan, Italy) and linearity was assessed in the range 0.05–5.00 µg/mL for all cannabinoids and also in the range 5.0–50 µg/mL for CBD in the case of CBD-rich, chemotype III, genotypes (R^2^ > 0.999). The total cannabinoid content was calculated according to Baratta et al. [37]. Three injections were performed for each sample and results are given as% (*w/w*), expressed as mean of replicates (*n* = 3). The limit of detection (LOD) was 0.001% and limit of quantification (LOQ) was 0.005%.

### 4.3. DNA Isolation and Genotyping PCR Analysis

For DNA isolation, 100 mg of frozen pooled leaves were finely ground by physical treatment with steel beads in a Tissue Lyser II (Qiagen) at 30 Hz for five minutes and extracted using the Invisorb^®^ Spin Plant Mini Kit (STRATEC molecular Gmbh, Berlin, Germany) according to the manufacturer’s instructions. DNA was eluted in 100 µL sterile water, quantified at the Infinite 200 PRO spectrophotometer (TECAN) and diluted to 10 ng/µL. 

The three-primers multiplex system used to genotype the *C. sativa* plants at the *B* locus [15] is detailed in Appendix A and generated either a 1081 bp (presence of a functional *CBDAS*), a 1192 bp (presence of a functional *THCAS*) or both fragments (presence of both functional genes).

### 4.4. RNA Isolation and cDNA Synthesis

For RNA isolation, 100 mg frozen samples were finely ground and extracted using the Spectrum Plant Total RNA Kit (Sigma Aldrich, Merck Life Science S.r.l., Milan, Italy). Total RNA was eluted in 50 μL of DEPC-treated water and spectrophotometrically quantified. Five hundred ng of total RNA were treated with 1 unit of DNAse I Amplification Grade (Sigma-Aldrich, Merk Life Science S.r.l., Milan, Italy) and retrotranscribed with the High-Capacity RNA to cDNA kit (Thermo Fisher Scientific, Waltham, MA, USA) according to manufacturer’s instructions. 

### 4.5. PCR Amplification, Cloning and Sequencing

*THCAS*- and *CBDAS*-specific primers from Onofri et al. [25], with some modifications as specified in Table 2, were used in this work to isolate the complete coding sequences of the genes.

For a specific amplification of the *CBCAS* genes, a search on *Cannabis* assemblies (available at: https://www.ncbi.nlm.nih.gov/assembly/?term=cannabis, accessed on 19 December 2019) was performed in order to identify conserved regions allowing the discrimination between active and “inactive” forms. Once identified, primers were designed using the software Primer3 [40,41].

The amplification reactions were performed on 20 ng of template DNA using the Invitrogen™ Platinum™ SuperFi™ polymerase (Thermo Fisher Scientific, Waltham, MA, USA), which has a reported fidelity >300 times higher than Taq, in 50 µL reaction. PCR products, purified using the NucleoSpin^®^ Gel and PCR Clean-up kit (Macherey-Nagel GmbH & Co. KG, D Düren, Germany), were cloned in pJET1.2 plasmid vectors (CloneJET PCR Cloning Kit, Thermo Fisher Scientific, Waltham, MA, USA) and transferred in *Escherichia coli* DH5α cells in accordance with the manufacturer’s protocol. Properly transformed cells were selected for ampicillin resistance (100 μg/mL final concentration) on LB -agar medium and colonies were screened by PCR, using the protocol and primers suggested in the cloning kit.

For each genotype, up to eight plasmids for each gene were isolated using the PureLink® Quick Plasmid Miniprep Kit (Thermo Fisher Scientific, Waltham, MA, USA). The inserts were then sequenced according to the Sanger method (BMR Genomics Srl, Padova, Italy), using the pJET 1.2 sequencing primers (pJET 1.2 forward: CGACTCACTATAGGGAGAGCGGC; pJET 1.2 reverse: AAGAACATCGATTTTCCATGGCAG). 

Only complete coding sequences were further considered in this work. The variability of *THCA* and *CBDA* synthases was evaluated firstly by aligning the sequences from each individual genotype in order to find unique single sequences. These were aligned with the two reference sequences of *THCA*- and *CBDA*-synthase (E33090 and E55107) and, finally, compared across the different genotypes. The sequence comparison at nucleotide and amino acid level was carried out using MUSCLE 3.8 (available at: https://www.ebi.ac.uk/Tools/msa/muscle/ accessed on 8 March 2021). For phylogenetic analysis the evolutionary history was inferred using the Neighbor-Joining method in the MEGA-X software [29,42]. The final figure of NJ DIRs tree was obtained by using the iTOL tree editor (The iTOL Platform). 

### 4.6. Transcriptional Analysis of Cannabinoid synthases

The transcriptional levels of the *CBDA-*, *THCA* and *CBCAS* and of three candidate reference genes *CsActin*, *CsRAN (Ras-related Nuclear protein)* and *CsClathrin* [43] were measured by reverse transcription quantitative real time PCR (RT-qPCR) using a Rotor-Gene 6000 (Corbett) and SYBR Green chemistry.

Each reaction contained 3 μL of a 1:9 dilution of cDNA, 5 μL of Power Up^®^SYBR master mix (Thermo Fisher Scientific), highly specific primers and RNA-free water to a final volume of 10 μL. Primer pairs, optimized conditions and annealing temperatures are listed in Table 3. Primer design and the optimization steps performed to assure the high specificity of amplification were performed following Pagliarani et al. [44] and a detailed description is reported in Appendix B. Further details of RT–qPCR conditions are reported in Supplementary File S2 following the Minimum Information for publication of Quantitative Real-Time PCR Experiments (MIQE) guidelines [45,46].

Different amplification conditions were used depending on the annealing temperature of the primers used: when this was below 60 °C, a two-step method was used, consisting of 15 s at variable temperature for annealing and 1 min at 72 °C for extension; when this was above 60 °C, a one-step method was used, consisting of 60 s at the desired temperature in which both annealing and extension are performed. Finally, a heat dissociation protocol (from 60 °C to 99 °C) was performed and a dissociation curve for each sample was generated. Three biological replicates were analyzed for each genotype, which in turn were tested in three technical replicates.

A standard curve was added in all assays, both for target and for reference genes. Standard curves were made of 5 points, prepared as four 1:4 serial dilutions of a 1:3 dilution of the cDNA (500 ng). 

To verify the specificity of reaction, each assay included also several negative controls as specified in Appendix B.

The amplification efficiency (E) of each primer pair was estimated using the slope of the regression line, according to the equation: E = 10^(−1/slope) − 1. The transcriptional level stability across samples of *CsActin*, *CsRAN* and *CsClathrin* was verified by software RefFinder [47] and, according to results, *CsClathrin* and *CsRAN* were selected as reference genes.

Raw data for target and reference genes were transformed using the ‘Standard Curve Method’; the transcripts level of target genes was normalized to the geometric mean of the transcripts level of *CsClathrin* and *CsRAN* and reported as Relative Quantitation (RQ) of transcriptional levels [48] expressed in Arbitrary Units (A.U.). Finally, the standard error of the mean of three biological replicates was calculated and reported in the graphs as error bar. Comparison between genotypes vs CINRO for each gene transcript levels was done using Student’s t-test with log-transformed expression data.

## 5. Conclusions

This work describes for the first time sequence variability of cannabinoid synthases coding sequences and their transcriptional profiles in a set of *Cannabis* genotypes, belonging to five different chemotypes. Highly specific primers were developed, to be used as robust markers for univocal identification and analysis of *THCAS*, *CBDAS* and *CBCAS* in different *Cannabis* tissues. While the higher levels of transcription detected for *THCAS* in respect to *CBDAS* can be related to the different end use of THCA-rich varieties compared with fiber varieties, the meaning of the low but almost constant level of transcription of *CBCAS* still remains to be fully understood. The existence of a cluster of *CBCA synthases* separated from functional *THCA synthases* suggest a different role in the plant as evolution and/or selection has created a whole set of highly conserved sequences. According to our data, *CBCAS* could play a role for producing residual THCA in CBD-predominant genotypes, but more research is still needed to confirm this hypothesis. 

Altogether, the results reported here and the assay developed will pave the way for more accurate functional studies *in planta* of these gene families and of how their reciprocal relationships can influence the quantitative component of chemotype.

## Figures and Tables

**Table 1:** Quantitative results of total cannabinoid analysis expressed as the mean of 3 replicates. LOD (limit of detection): 0.001%. LOQ (limit of quantification): 0.005%. Information regarding inflorescences sampling period is expressed as days after sowing for hemp varieties, as days from onset flowering for CINBOL and CINRO. Results of genotyping obtained using the marker B1080/B1192 are given for each genotype. *B*_D_ and *B*_T_ refer to the allelic status as determined by the marker [25]. Data are expressed as percent of total inflorescence dry weight.

Genotype	Sampling	CBD (%)	CBG (%)	THC (%)	CBC (%)	Marker Phenotype
Santhica 27 ^a^	123	0.26	1.55	0.04	<LOD	*B_D_/B_D_*
Carmagnola	151	5.32	0.28	0.16	<LOQ	*B_D_/B_D_*
Bernabeo	151	0.74	2.69	0.04	<LOQ	*B_D_/B_D_*
Carmaleonte	123	1.92	0.10	0.10	<LOQ	*B_D_/B_D_*
CS	151	5.49	0.20	0.17	<LOQ	*B_D_/B_D_*
Ermo	151	0.05	<LOD	<LOD	<LOD	*B_D_/B_D_*
Fibrante	151	3.61	0.09	0.13	<LOQ	*B_D_/B_D_*
Fibranova	151	2.34	0.10	0.07	<LOQ	*B_D_/B_D_*
Eletta Campana	151	3.85	0.17	0.11	<LOQ	*B_D_/B_D_*
Codimono	123	4.02	0.15	0.14	<LOQ	*B_D_/B_D_*
Futura 75 ^a^	123	2.34	0.12	0.07	<LOD	*B_D_/B_D_*
CINBOL	50	0.01	0.37	3.71	0.01	*B_T_/B_T_*
CINRO	50	2.43	0.27	1.57	0.02	*B_T_/B_D_*

^a^ French varieties.

**Table 2:** List of primer pairs used for full length coding sequences (cds) amplification. Forward (Fw) and Reverse (Rv) primer sequences, amplicon length (bp) and references are given. Start and stop codons are underlined for each primer.

Gene	Primer Sequence (5′–3′)	Amplicon Length (bp)	Reference
*CBCAS-cds*	Fw: TGAAGAAAAATGAATTGCTCAACATTC	1666	This work
	Rv: ACATAGTATGGGTAGATAATTAATGATGAC		
*THCAS-cds*	Fw: ATGAATTGCTCAGCATTTTCCTT	1635	[25]
	Rv: ATGATGATGCGGTGGAAGA ^a^		
*CBDAS-cds*	Fw: ATGAAGTGCTCAACATTCTCCTT	1635	revised from [25]
	Rv: TTAATGACGATGCCGTGGAA		

^a^ the stop codon is immediately after the end of the reverse primer.

**Table 3:** List of primer pairs used for RT-qPCR. Forward (Fw) and reverse (Rv) primer sequences, amplicon length expressed in base pairs (bp), regression coefficient (R^2^), PCR efficiency (Eff), annealing temperature (Ta) and usage concentrations (μM) are given.

Gene	Primer Sequence (5′–3′)	Amplicon Length (bp)	R^2^	Eff (%)	Ta	[μM]
*CBDAS*-RTqPCR	Fw: GCAATACACACTTACTTCTCTTCAGTTTTC	241	0.99	1.04	61.5	0.075
	Rv: ACGTAGTCTAACTTATCTTGAAAGCAC					
*THCAS*-RTqPCR	Fw: AAAACTTCCTTAAATGCTTCTCAA	198	0.94	0.8	58	0.175
	Rv: TAAAATAGTTGCTTGGATATGGGAGTT					
*CBCAS*-RTqPCR	Fw: GCTCACGACTCACTTCAGAACTAG	198	0.98	1.01	62	0.1
	Rv: GTAGAAGATGGTTGTATCAATCCAGCTC					

**Figure A1:**
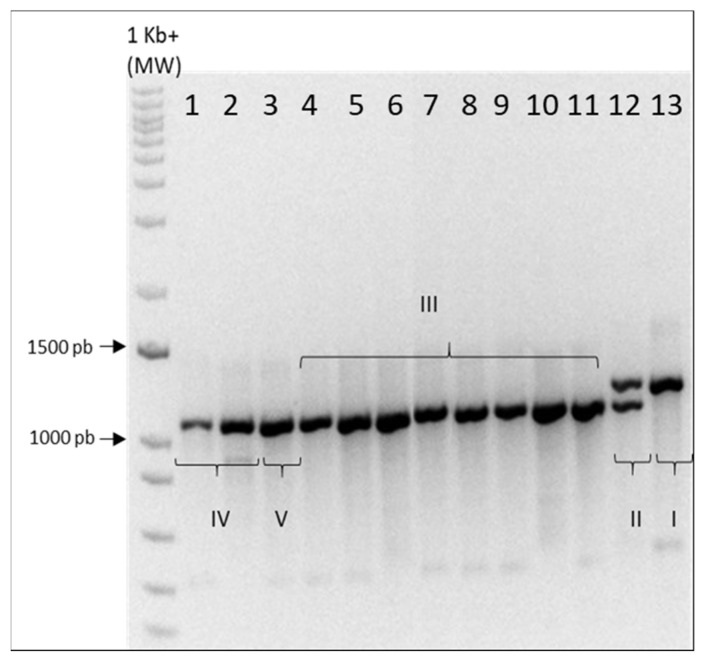
Chemotype determination. The respective chemotypes are indicated for each genotype (I–V). From left to right the genotypes are: 1, Santhica 27; 2, Bernabeo; 3, Ermo; 4, Fibranova; 5, Eletta Campana; 6, Futura 75; 7, Carmagnola; 8, Carmaleonte; 9, Codimono; 10, C.S.; 11, Fibrante; 12, CINRO; 13, CINBOL.

**Figure A2:**
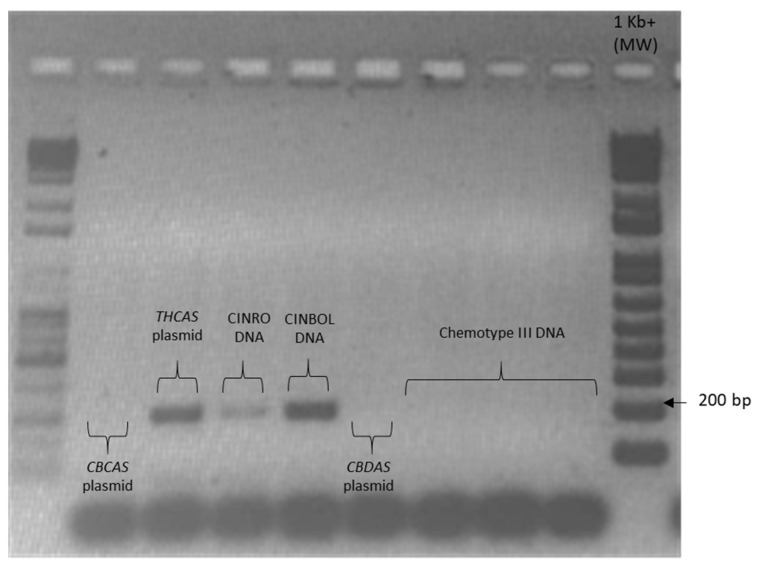
Specificity of primers for *THCAS*. The expected amplification band of 198 bp was obtained from pJET1.2 containing the *THCAS* and DNA of CINRO and CINBOL. Amplification was not obtained from pJET1.2 plasmid containing the *CBCAS*, and the *CBDAS*, and neither using DNA from hemp varieties as template.

**Figure A3:**
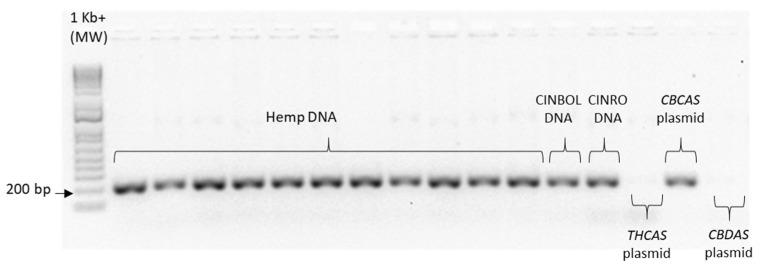
Specificity of primers for *CBCAS* genes. The expected amplification band of 198 bp was obtained from pJET1.2 containing the CBCAS and DNA of all tested genotypes. Amplification was not obtained from pJET1.2 plasmid containing the *THCAS* and the *CBDAS*, indicating a great specificity of this primer pairs.

**Figure A4:**
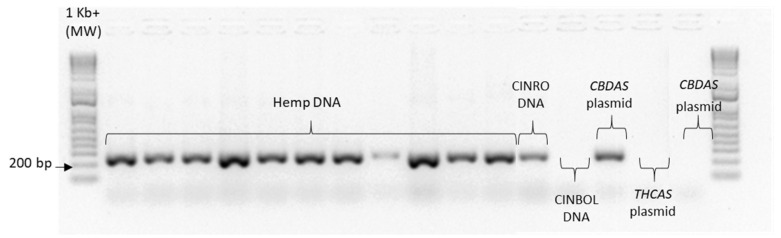
Specificity of primers for *CBDAS*. The expected amplification band of 241 bp was obtained from pJET1.2 containing the CBDAS and DNA from hemp and from CINRO. Amplification was not obtained from pJET1.2 plasmid containing the *THCAS* and *CBCAS*. Primers work only on functional CBDAS sequences.

**Figure A5:**
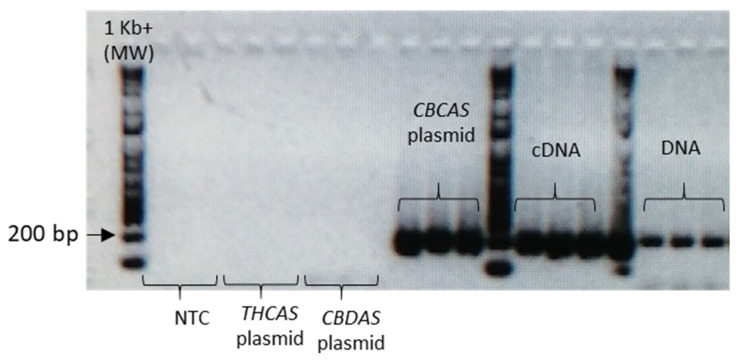
Agarose gel electrophoresis results of *CBCAS* primers amplification on plasmids containing each cannabinoid synthases, genomic DNA and cDNA of a hemp plant in triplicate. The PCR assays contained also 2 negative controls, where water was added instead of template (lane 1 and 2).

**Figure A6:**
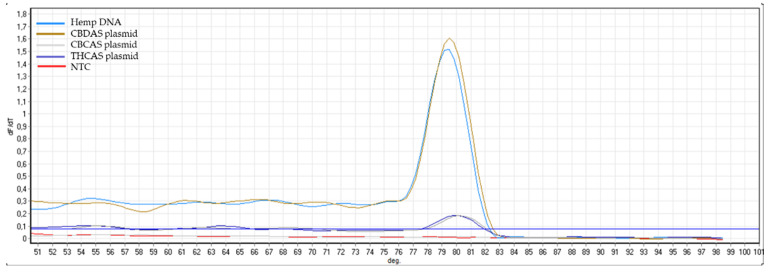
Melting curve plots, which display data collected during a melting curve stage with primers CBDAS_F and CBDAS_R. Peaks in the melting curve may indicate the melting temperature (Tm) of a target or identify nonspecific PCR amplification. No peaks were seen for the no template control (H_2_O) and for negative controls (*THCAS* plasmid and *CBCAS* plasmid).

**Figure A7:**
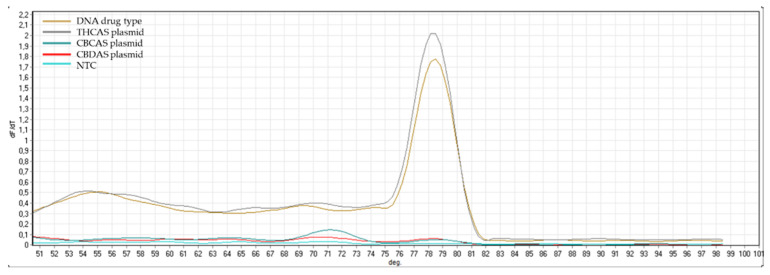
Melting curve plots, which display data collected during a melting curve stage with primers THCAS_F and THCAS_R. Peaks in the melting curve may indicate the melting temperature (Tm) of a target or identify nonspecific PCR amplification. No peaks were observed for the no template control (H_2_O) and for negative controls (*CBDAS* plasmid and *CBCAS* plasmid).

**Figure A8:**
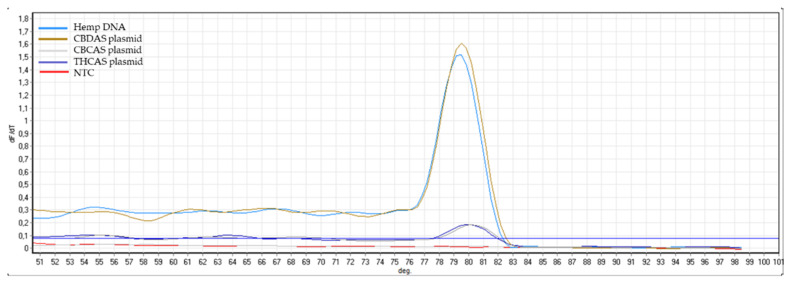
Melting curve plots, which display data collected during a melting curve stage with primers CBCAS_F and CBCAS_R. Peaks in the melting curve may indicate the melting temperature (Tm) of a target or identify nonspecific PCR amplification. No peaks were seen for the no template control (H_2_O) and for negative controls (*THCAS* plasmid, *CBDAS* plasmid).

## Data Availability

Data is contained within the article and Supplementary Materials.

## Supplementary Materials

The following are available online at https://www.mdpi.com/article/10.3390/plants10091857/s1, Supplementary File S1: Alignment of *CBCAS* nucleotide sequences; Supplementary File S2: detailed MIQE checklist; Supplementary Tables S1–S4; Table S1: Summary of features of the *Cannabis sativa* genomes accessed, Table S2: Sequence variation within *Cannabis* germplasm, Table S3: SNPs found in the *CBCAS* sequences found in *Cannabis* germplasm, Table S4: SNPs found in the *CBDAS* sequences in *Cannabis* germplasm, Table S5: Ratio between *CBDAS* and *CBCAS* relative quantitation.

## Appendix A

Chemotype assessment.

**Table A1:** Primer sequences used for chemotype assessment.

Primer Name	Sequence (5′->3′)
THCAS/CBDAS fw	AAGAAAGTTGGCTTGCAG
CBDAS rev	ATCCAGTTTAGATGCTTTTCGT
THCAS rev	TTAGGACTCGCATGATTAGTTTTTC

**Table A2:** PCR reaction set up for three-primers marker system.

Component	Final Concentration
Water	Up to 25 µL
10× PCR Buffer, -Mg	1×
50 mM MgCl2	1.5 mM
10 mM dNTP Mix	0.2 mM each
10 µM forward primer	0.2 µM
10 µM reverse primer 1	0.2 µM
10 µM reverse primer 2	0.2 µM
Template DNA	10 ng
*Taq* DNA Polymerase (5U/µL)	0.04 U/µL

**Table A3:** PCR program for three-primers marker system.

Step	Temperature	Time	Cycles
Initial denaturation	94 °C	3 min	1
Denaturation	94 °C	45 s	32
Annealing	62 °C	1 min	
Extension	72 °C	2 min	
Final extension	72 °C	10 min	1

The chemotype assessment was performed by molecular markers described by Pacifico et al. [15] in order to confirm the chemotypes of the 13 genotypes.

The amplification reaction, consisting of 10 ng of DNA and the recombinant Taq DNA Polymerase (Invitrogen), was performed in a T-Gradient Thermocycler (Biometra) in a final volume of 25 µL.

5 µL of PCR products were checked after adding 1 µL of 6× DNA loading dye (Thermo Fisher Scientific) by electrophoresis on 1% agarose gel (TAE buffer 1×), stained with Gel Red (Biotum). 1Kb plus DNA Ladder (Invitrogen) was used as marker for amplicon size. DNA was visualized under UV light on a transilluminator and digitally photographed with AlphaImager HP (Alpha Innotech, Santa Clara, CA, USA).

## Appendix B

Optimization of RT-qPCR primers for selective and quantitative amplification of target *cannabinoid synthase* genes.

Primer design and amplification reactions were optimized to distinguish the different cannabinoid synthases sequences, developing highly specific assays for gene detection and transcriptional analyses.

Gene-specific primer pairs were designed using the software Primer3. Since the different genes encoding for Cannabinoid Synthases share high similarity at the nucleotide level, primers were carefully designed in presence of SNPs differentiating genes, identified by sequences alignment, by keeping the SNPs at the 3’ end of the primers. The primer pairs designed and employed in this work are shown in Table 3 of the main text.

Primer specificity was verified in different ways as described elsewhere [44]. First, primer pairs were searched by BlastN against *Cannabis sativa* non-redundant database at NCBI. Second, the presence of a single PCR product after end-point PCR and the absence of non-target amplification were assessed. The PCR reactions were performed in a 10 μL total volume, with 200 μM dNTPs, 0.2 U Phusion DNA Polymerase (Thermo Scientific) and different concentrations of the specific primer pairs. As template, 0.5 pg of plasmid DNA, 1.5 μL of cDNA (diluted 1:10, corresponding to 2.5 ng starting RNA) and 10 ng DNA were used.

Different pJET1.2 plasmid vectors (CloneJET PCR Cloning Kit, Thermo Scientific) obtained as described in paragraph 2.5 of the main text, were used in this second step: one containing the specific gene targeted by the primer pair (positive control), and one or more plasmids containing other cannabinoids synthase gene sequences (negative controls).

PCR were performed following this condition: 98 °C for 30 s, 30 cycles of 10 s at 98 °C, 15 s at variable temperatures, and 15 s at 72 °C, followed by a final extension of 5 min at 72 °C. In order to optimize the reaction to get a specific product, different primers concentrations and annealing temperature were tested until the best condition was found.

The reaction was considered optimized only when amplification band was present in the positive control and absent in the negative ones (Figure A2, Figure A3 and Figure A4).

Primer pairs can be used as markers on DNA or to verify if the specific target gene is expressed using cDNA as template, as indicated in Figure A5.

Finally, primers specificity was tested by RT-qPCR with a RotorGene 6000 (Corbett). In this specificity assay, two parameters were changed to optimize the amplification conditions: the annealing temperature (from 58 to 62 °C) and the primers concentration (from 75 nM to 1750 nM). Amplification reactions were conducted in a final volume of 10 μL containing the Power Up^®^ SYBR master mix (Thermo Fisher Scientific), primer pair (different concentrations) and RNase-free water. As template, cDNA, DNA and plasmid DNA were used as indicated above.

Different amplification conditions were used depending on the annealing temperature of the primers used: when this was below 60 °C, a two-step method was used, consisting of 15 s at variable temperature for annealing and 1 min at 72 °C for extension; when this was above 60 °C, a one-step method was used, consisting of 60 s at the desired temperature in which both annealing and extension are performed. Finally, to ensure the absence of not-specific PCR products and primer dimers, a heat dissociation protocol (from 60 °C to 99 °C) was performed and a dissociation curve for each sample was generated. Primers were considered as specific when they amplify a positive control and give no signal for negative controls (in both cases the controls are represented by plasmids with a known sequence). The production of a single melting curve peak in positive controls and samples suggested that the amplicon was specific (Figure A6, Figure A7 and Figure A8).
