# Supplementary material for: Analysis of Sequence Variability and Transcriptional Profile of Cannabinoid synthase Genes in Cannabis sativa L. Chemotypes with a Focus on Cannabichromenic acid synthase
Source: Plants (Basel). 2021 Sep 8;10(9):1857. doi: 10.3390/plants10091857 (PMC8466818; doi:10.3390/plants10091857)
Supplement: Supplementary file 1 [file plants-10-01857-s001.zip › Supplementary File 1.pdf]

Supplementary File 1

Analysis of sequence variability and transcriptional profile of *cannabinoid synthase* genes in *Cannabis sativa* L. chemotypes with a focus on *cannabichromenic acid synthase*

Flavia Fulvio[1,2], Roberta Paris[1*], Massimo Montanari[1], Cinzia Citti[3,4], Vincenzo Cilento[1], Laura Bassolino[1], Anna Moschella[1], Ilaria Alberti[5], Nicola Pecchioni[6], Giuseppe Cannazza[3,4] and Giuseppe Mandolino[1]

1 CREA-Research Center for Cereal and Industrial Crops, Via di Corticella 133, 40128 Bologna, Italy

2 Department of Sciences of Agriculture, Food Natural resources and Engineering, University of Foggia, Via Napoli 25, 71122 Foggia, Italy

3 CNR NANOTEC – Institute of Nanotechnology, Via Monteroni, 73100, Lecce, Italy

4 Department of Life Science, University of Modena and Reggio Emilia, Via G. Campi 103, 41125, Modena, Italy

5 CREA-Research Center for Cereal and Industrial Crops, Via G. Amendola 82, 45100 Rovigo, Italy

6 CREA-Research Center for Cereal and Industrial Crops, S.S. 673 Km 25,200, 71122, Foggia, Italy

* Correspondence: roberta.paris@crea.gov.it

Supplementary File S1: nucleotide sequence alignment of *CBCAS* genes downloaded from sequenced genomes, AB212830 and a *CBCAS* sequenced from one of the genotypes under study.

CLUSTAL multiple sequence alignment by MUSCLE (3.8)

```
AB212830.1                         ATGAATTGCTCAGCATTTTCCTTTTGGTTTGTTTGCAAAATAATATTTTTCTTTCTCTCA
NC_044378.1:25821957-25823591      ATGAATTGCTCAACATTCTCCTTTTGGTTTGTTTGCAAAATAATATTTTTCTTTCTCTCA
QKVJ02004887.1:13943-15577         ATGAATTGCTCAACATTCTCCTTTTGGTTTGTTTGCAAAATAATATTTTTCTTTCTCTCA
MNPR01002882.1:12890-14524         ATGAATTGCTCAACATTCTCCTTTTGGTTTGTTTGCAAAATAATATTTTTCTTTCTCTCA
MXBD01006695.1:30197-31831         ATGAATTGCTCAACATTCTCCTTTTGGTTTGTTTGCAAAATAATATTTTTCTTTCTCTCA
AGQN03005496.1:2986-4620           ATGAATTGCTCAACATTCTCCTTTTGGTTTGTTTGCAAAATAATATTTTTCTTTCTCTCA
QVPT02000161.1:1531540-1533174     ATGAATTGCTCAACATTCTCCTTTTGGTTTGTTTGCAAAATAATATTTTTCTTTCTCTCA
MW429551                           ATGAATTGCTCAACATTCTCCTTTTGGTTTGTTTGCAAAATAATATTTTTCTTTCTCTCA
                                   ************ **** ******************************************

AB212830.1                         TTCAATATCCAAATTTCAATAGCTAATCCTCAAGAAAACTTCCTTAAATGCTTCTCGGAA
NC_044378.1:25821957-25823591      TTCAATATCCAAATTTCAATAGCTAATCCTCAAGAAAACTTCCTTAAATGCTTCTCGGAA
QKVJ02004887.1:13943-15577         TTCAATATCCAAATTTCAATAGCTAATCCTCAAGAAAACTTCCTTAAATGCTTCTCGGAA
MNPR01002882.1:12890-14524         TTCAATATCCAAATTTCAATAGCTAATCCTCAAGAAAACTTCCTTAAATGCTTCTCGGAA
MXBD01006695.1:30197-31831         TTCAATATCCAAATTTCAATAGCTAATCCTCAAGAAAACTTCCTTAAATGCTTCTCGGAA
AGQN03005496.1:2986-4620           TTCAATATCCAAATTTCAATAGCTAATCCTCAAGAAAACTTCCTTAAATGCTTCTCGGAA
QVPT02000161.1:1531540-1533174     TTCAATATCCAAATTTCAATAGCTAATCCTCAAGAAAACTTCCTTAAATGCTTCTCGGAA
MW429551                           TTCAATATCCAAATTTCAATAGCTAATCCTCAAGAAAACTTCCTTAAATGCTTCTCGGAA
                                   ************************************************************

AB212830.1                         TATATTCCTAACAATCCAGCAAATCCAAAATTCATATACACTCAACACGACCAATTGTAT
NC_044378.1:25821957-25823591      TATATTCCTAACAATCCAGCAAATCCAAAATTCATATACACTCAACACGACCAATTGTAT
QKVJ02004887.1:13943-15577         TATATTCCTAACAATCCAGCAAATCCAAAATTCATATACACTCAACACGACCAATTGTAT
MNPR01002882.1:12890-14524         TATATTCCTAACAATCCAGCAAATCCAAAATTCATATACACTCAACACGACCAATTGTAT
MXBD01006695.1:30197-31831         TATATTCCTAACAATCCAGCAAATCCAAAATTCATATACACTCAACACGACCAATTGTAT
AGQN03005496.1:2986-4620           TATATTCCTAACAATCCAGCAAATCCAAAATTCATATACACTCAACACGACCAATTGTAT
QVPT02000161.1:1531540-1533174     TATATTCCTAACAATCCAGCAAATCCAAAATTCATATACACTCAACACGACCAATTGTAT
MW429551                           TATATTCCTAACAATCCAGCAAATCCAAAATTCATATACACTCAACACGACCAATTGTAT
                                   ************************************************************

AB212830.1                         ATGTCTGTCCTGAATTCGACAATACAAAATCTTAGATTCACCTCTGATACAACCCCAAAA
NC_044378.1:25821957-25823591      ATGTCTGTCCTGAATTCGACAATACAAAATCTTAGATTCACCTCTGATACAACCCCAAAA
QKVJ02004887.1:13943-15577         ATGTCTGTCCTGAATTCGACAATACAAAATCTTAGATTCACCTCTGATACAACCCCAAAA
MNPR01002882.1:12890-14524         ATGTCTGTCCTGAATTCGACAATACAAAATCTTAGATTCACCTCTGATACAACCCCAAAA
MXBD01006695.1:30197-31831         ATGTCTGTCCTGAATTCGACAATACAAAATCTTAGATTCACCTCTGATACAACCCCAAAA
AGQN03005496.1:2986-4620           ATGTCTGTCCTGAATTCGACAATACAAAATCTTAGATTCACCTCTGATACAACCCCAAAA
QVPT02000161.1:1531540-1533174     ATGTCTGTCCTGAATTCGACAATACAAAATCTTAGATTCACCTCTGATACAACCCCAAAA
MW429551                           ATGTCTGTCCTGAATTCGACAATACAAAATCTTAGATTCACCTCTGATACAACCCCAAAA
                                   ************************************************************

AB212830.1                         CCACTCGTTATTGTCACTCCTTCAAATGTCTCCCATATCCAGGCCAGTATTCTCTGCTCC
NC_044378.1:25821957-25823591      CCACTCGTTATTGTCACTCCTTCAAATGTCTCCCATATCCAGGCCAGTATTCTCTGCTCC
QKVJ02004887.1:13943-15577         CCACTCGTTATTGTCACTCCTTCAAATGTCTCCCATATCCAGGCCAGTATTCTCTGCTCC
MNPR01002882.1:12890-14524         CCACTCGTTATTGTCACTCCTTCAAATGTCTCCCATATCCAGGCCAGTATTCTCTGCTCC
MXBD01006695.1:30197-31831         CCACTCGTTATTGTCACTCCTTCAAATGTCTCCCATATCCAGGCCAGTATTCTCTGCTCC
AGQN03005496.1:2986-4620           CCACTCGTTATTGTCACTCCTTCAAATGTCTCCCATATCCAGGCCAGTATTCTCTGCTCC
QVPT02000161.1:1531540-1533174     CCACTCGTTATTGTCACTCCTTCAAATGTCTCCCATATCCAGGCCAGTATTCTCTGCTCC
MW429551                           CCACTCGTTATTGTCACTCCTTCAAATGTCTCCCATATCCAGGCCAGTATTCTCTGCTCC
                                   ************************************************************

AB212830.1                         AAGAAAGTTGGTTTGCAGATTCGAACTCGAAGCGGTGGCCATGATGCTGAGGGTTTGTCC
NC_044378.1:25821957-25823591      AAGAAAGTTGGTTTGCAGATTCGAACTCGAAGCGGTGGCCATGATGCTGAGGGTTTGTCC
QKVJ02004887.1:13943-15577         AAGAAAGTTGGTTTGCAGATTCGAACTCGAAGCGGTGGCCATGATGCTGAGGGTTTGTCC
MNPR01002882.1:12890-14524         AAGAAAGTTGGTTTGCAGATTCGAACTCGAAGCGGTGGCCATGATGCTGAGGGTTTGTCC
MXBD01006695.1:30197-31831         AAGAAAGTTGGTTTGCAGATTCGAACTCGAAGCGGTGGCCATGATGCTGAGGGTTTGTCC
AGQN03005496.1:2986-4620           AAGAAAGTTGGTTTGCAGATTCGAACTCGAAGCGGTGGCCATGATGCTGAGGGTTTGTCC
QVPT02000161.1:1531540-1533174     AAGAAAGTTGGTTTGCAGATTCGAACTCGAAGCGGTGGCCATGATGCTGAGGGTTTGTCC
MW429551                           AAGAAAGTTGGTTTGCAGATTCGAACTCGAAGCGGTGGCCATGATGCTGAGGGTTTGTCC
                                   ************************************************************

AB212830.1                         TACATATCTCAAGTCCCATTTGCTATAGTAGACTTGAGAAACATGCATACGGTCAAAGTA
NC_044378.1:25821957-25823591      TACATATCTCAAGTCCCATTTGCTATAGTAGACTTGAGAAACATGCATACGGTCAAAGTA
QKVJ02004887.1:13943-15577         TACATATCTCAAGTCCCATTTGCTATAGTAGACTTGAGAAACATGCATACGGTCAAAGTA
MNPR01002882.1:12890-14524         TACATATCTCAAGTCCCATTTGCTATAGTAGACTTGAGAAACATGCATACGGTCAAAGTA
MXBD01006695.1:30197-31831         TACATATCTCAAGTCCCATTTGCTATAGTAGACTTGAGAAACATGCATACGGTCAAAGTA
AGQN03005496.1:2986-4620           TACATATCTCAAGTCCCATTTGCTATAGTAGACTTGAGAAACATGCATACGGTCAAAGTA
QVPT02000161.1:1531540-1533174     TACATATCTCAAGTCCCATTTGCTATAGTAGACTTGAGAAACATGCATACGGTCAAAGTA
MW429551                           TACATATCTCAAGTCCCATTTGCTATAGTAGACTTGAGAAACATGCATACGGTCAAAGTA
                                   ************************************************************

AB212830.1                         GATATTCATAGCCAAACTGCGTGGGTTGAAGCCGGAGCTACCCTTGGAGAAGTTTATTAT
```

```
NC_044378.1:25821957-25823591      GATATTCATAGCCAAACTGCGTGGGTTGAAGCCGGAGCTACCCTTGGAGAAGTTTATTAT
QKVJ02004887.1:13943-15577         GATATTCATAGCCAAACTGCGTGGGTTGAAGCCGGAGCTACCCTTGGAGAAGTTTATTAT
MNPR01002882.1:12890-14524         GATATTCATAGCCAAACTGCGTGGGTTGAAGCCGGAGCTACCCTTGGAGAAGTTTATTAT
MXBD01006695.1:30197-31831         GATATTCATAGCCAAACTGCGTGGGTTGAAGCCGGAGCTACCCTTGGAGAAGTTTATTAT
AGQN03005496.1:2986-4620           GATATTCATAGCCAAACTGCGTGGGTTGAAGCCGGAGCTACCCTTGGAGAAGTTTATTAT
QVPT02000161.1:1531540-1533174     GATATTCATAGCCAAACTGCGTGGGTTGAAGCCGGAGCTACCCTTGGAGAAGTTTATTAT
MW429551                           GATATTCATAGCCAAACTGCGTGGGTTGAAGCCGGAGCTACCCTTGGAGAAGTTTATTAT
                                   ************************************************************

AB212830.1                         TGGATCAATGAGATGAATGAGAATTTTAGTTTTCCTGGTGGGTATTGCCCTACTGTTGGC
NC_044378.1:25821957-25823591      TGGATCAATGAGATGAATGAGAATTTTAGTTTTCCTGGTGGGTATTGCCCTACTGTTGGC
QKVJ02004887.1:13943-15577         TGGATCAATGAGATGAATGAGAATTTTAGTTTTCCTGGTGGGTATTGCCCTACTGTTGGC
MNPR01002882.1:12890-14524         TGGATCAATGAGATGAATGAGAATTTTAGTTTTCCTGGTGGGTATTGCCCTACTGTTGGC
MXBD01006695.1:30197-31831         TGGATCAATGAGATGAATGAGAATTTTAGTTTTCCTGGTGGGTATTGCCCTACTGTTGGC
AGQN03005496.1:2986-4620           TGGATCAATGAGATGAATGAGAATTTTAGTTTTCCTGGTGGGTATTGCCCTACTGTTGGC
QVPT02000161.1:1531540-1533174     TGGATCAATGAGATGAATGAGAATTTTAGTTTTCCTGGTGGGTATTGCCCTACTGTTGGC
MW429551                           TGGATCAATGAGATGAATGAGAATTTTAGTTTTCCTGGTGGGTATTGCCCTACTGTTGGC
                                   ************************************************************

AB212830.1                         GTAGGTGGACACTTTAGTGGAGGAGGCTATGGAGCATTGATGCGAAATTATGGCCTTGCG
NC_044378.1:25821957-25823591      GTAGGTGGACACTTTAGTGGAGGAGGCTATGGAGCATTGATGCGAAATTATGGCCTTGCG
QKVJ02004887.1:13943-15577         GTAGGTGGACACTTTAGTGGAGGAGGCTATGGAGCATTGATGCGAAATTATGGCCTTGCG
MNPR01002882.1:12890-14524         GTAGGTGGACACTTTAGTGGAGGAGGCTATGGAGCATTGATGCGAAATTATGGCCTTGCG
MXBD01006695.1:30197-31831         GTAGGTGGACACTTTAGTGGAGGAGGCTATGGAGCATTGATGCGAAATTATGGCCTTGCG
AGQN03005496.1:2986-4620           GTAGGTGGACACTTTAGTGGAGGAGGCTATGGAGCATTGATGCGAAATTATGGCCTTGCG
QVPT02000161.1:1531540-1533174     GTAGGTGGACACTTTAGTGGAGGAGGCTATGGAGCATTGATGCGAAATTATGGCCTTGCG
MW429551                           GTAGGTGGACACTTTAGTGGAGGAGGCTATGGAGCATTGATGCGAAATTATGGCCTTGCG
                                   ************************************************************

AB212830.1                         GCTGATAATATCATTGATGCACACTTAGTCAATGTTGATGGAAAAGTTCTAGATCGAAAA
NC_044378.1:25821957-25823591      GCTGATAATATCATTGATGCACACTTAGTCAATGTTGATGGAAAAGTTCTAGATCGAAAA
QKVJ02004887.1:13943-15577         GCTGATAATATCATTGATGCACACTTAGTCAATGTTGATGGAAAAGTTCTAGATCGAAAA
MNPR01002882.1:12890-14524         GCTGATAATATCATTGATGCACACTTAGTCAATGTTGATGGAAAAGTTCTAGATCGAAAA
MXBD01006695.1:30197-31831         GCTGATAATATCATTGATGCACACTTAGTCAATGTTGATGGAAAAGTTCTAGATCGAAAA
AGQN03005496.1:2986-4620           GCTGATAATATCATTGATGCACACTTAGTCAATGTTGATGGAAAAGTTCTAGATCGAAAA
QVPT02000161.1:1531540-1533174     GCTGATAATATCATTGATGCACACTTAGTCAATGTTGATGGAAAAGTTCTAGATCGAAAA
MW429551                           GCTGATAATATCATTGATGCACACTTAGTCAATGTTGATGGAAAAGTTCTAGATCGAAAA
                                   ************************************************************

AB212830.1                         TCCATGGGAGAAGATCTATTTTGGGCTATACGTGGTGGAGGAGGAGAAAACTTTGGAATC
NC_044378.1:25821957-25823591      TCCATGGGAGAAGATCTATTTTGGGCTATACGTGGTGGAGGAGGAGAAAACTTTGGAATC
QKVJ02004887.1:13943-15577         TCCATGGGAGAAGATCTATTTTGGGCTATACGTGGTGGAGGAGGAGAAAACTTTGGAATC
MNPR01002882.1:12890-14524         TCCATGGGAGAAGATCTATTTTGGGCTATACGTGGTGGAGGAGGAGAAAACTTTGGAATC
MXBD01006695.1:30197-31831         TCCATGGGAGAAGATCTATTTTGGGCTATACGTGGTGGAGGAGGAGAAAACTTTGGAATC
AGQN03005496.1:2986-4620           TCCATGGGAGAAGATCTATTTTGGGCTATACGTGGTGGAGGAGGAGAAAACTTTGGAATC
QVPT02000161.1:1531540-1533174     TCCATGGGAGAAGATCTATTTTGGGCTATACGTGGTGGAGGAGGAGAAAACTTTGGAATC
MW429551                           TCCATGGGAGAAGATCTATTTTGGGCTATACGTGGTGGAGGAGGAGAAAACTTTGGAATC
                                   ************************************************************

AB212830.1                         ATTGCAGCATGGAAAATCAAACTTGTTGTTGTCCCATCAAAGGCTACTATATTCAGTGTT
NC_044378.1:25821957-25823591      ATTGCAGCATGGAAAATCAAACTTGTTGTTGTCCCATCAAAGGCTACTATATTCAGTGTT
QKVJ02004887.1:13943-15577         ATTGCAGCATGGAAAATCAAACTTGTTGTTGTCCCATCAAAGGCTACTATATTCAGTGTT
MNPR01002882.1:12890-14524         ATTGCAGCATGGAAAATCAAACTTGTTGTTGTCCCATCAAAGGCTACTATATTCAGTGTT
MXBD01006695.1:30197-31831         ATTGCAGCATGGAAAATCAAACTTGTTGTTGTCCCATCAAAGGCTACTATATTCAGTGTT
AGQN03005496.1:2986-4620           ATTGCAGCATGGAAAATCAAACTTGTTGTTGTCCCATCAAAGGCTACTATATTCAGTGTT
QVPT02000161.1:1531540-1533174     ATTGCAGCATGGAAAATCAAACTTGTTGTTGTCCCATCAAAGGCTACTATATTCAGTGTT
MW429551                           ATTGCAGCATGGAAAATCAAACTTGTTGTTGTCCCATCAAAGGCTACTATATTCAGTGTT
                                   ************************************************************

AB212830.1                         AAAAAGAACATGGAGATACATGGGCTTGTCAAGTTATTTAACAAATGGCAAAATATTGCT
NC_044378.1:25821957-25823591      AAAAAGAACATGGAGATACATGGGCTTGTCAAGTTATTTAACAAATGGCAAAATATTGCT
QKVJ02004887.1:13943-15577         AAAAAGAACATGGAGATACATGGGCTTGTCAAGTTATTTAACAAATGGCAAAATATTGCT
MNPR01002882.1:12890-14524         AAAAAGAACATGGAGATACATGGGCTTGTCAAGTTATTTAACAAATGGCAAAATATTGCT
MXBD01006695.1:30197-31831         AAAAAGAACATGGAGATACATGGGCTTGTCAAGTTATTTAACAAATGGCAAAATATTGCT
AGQN03005496.1:2986-4620           AAAAAGAACATGGAGATACATGGGCTTGTCAAGTTATTTAACAAATGGCAAAATATTGCT
QVPT02000161.1:1531540-1533174     AAAAAGAACATGGAGATACATGGGCTTGTCAAGTTATTTAACAAATGGCAAAATATTGCT
MW429551                           AAAAAGAACATGGAGATACATGGGCTTGTCAAGTTATTTAACAAATGGCAAAATATTGCT
                                   ************************************************************

AB212830.1                         TACAAGTATGACAAAGATTTAATGCTCACGACTCACTTCAGAACTAGGAATATTACAGAT
NC_044378.1:25821957-25823591      TACAAGTATGACAAAGATTTAATGCTCACGACTCACTTCAGAACTAGGAATATTACAGAT
QKVJ02004887.1:13943-15577         TACAAGTATGACAAAGATTTAATGCTCACGACTCACTTCAGAACTAGGAATATTACAGAT
MNPR01002882.1:12890-14524         TACAAGTATGACAAAGATTTAATGCTCACGACTCACTTCAGAACTAGGAATATTACAGAT
MXBD01006695.1:30197-31831         TACAAGTATGACAAAGATTTAATGCTCACGACTCACTTCAGAACTAGGAATATTACAGAT
AGQN03005496.1:2986-4620           TACAAGTATGACAAAGATTTAATGCTCACGACTCACTTCAGAACTAGGAATATTACAGAT
QVPT02000161.1:1531540-1533174     TACAAGTATGACAAAGATTTAATGCTCACGACTCACTTCAGAACTAGGAATATTACAGAT
MW429551                           TACAAGTATGACAAAGATTTAATGCTCACGACTCACTTCAGAACTAGGAATATTACAGAT
                                   ************************************************************
```

```
AB212830.1                          AATCATGGGAAGAATAAGACTACAGTACATGGTTACTTCTCTTCCATTTTTCTTGGTGGA
NC_044378.1:25821957-25823591       AATCATGGGAAGAATAAGACTACAGTACATGGTTACTTCTCTTCCATTTTTCTTGGTGGA
QKVJ02004887.1:13943-15577          AATCATGGGAAGAATAAGACTACAGTACATGGTTACTTCTCTTCCATTTTTCTTGGTGGA
MNPR01002882.1:12890-14524          AATCATGGGAAGAATAAGACTACAGTACATGGTTACTTCTCTTCCATTTTTCTTGGTGGA
MXBD01006695.1:30197-31831          AATCATGGGAAGAATAAGACTACAGTACATGGTTACTTCTCTTCCATTTTTCTTGGTGGA
AGQN03005496.1:2986-4620            AATCATGGGAAGAATAAGACTACAGTACATGGTTACTTCTCTTCCATTTTTCTTGGTGGA
QVPT02000161.1:1531540-1533174      AATCATGGGAAGAATAAGACTACAGTACATGGTTACTTCTCTTCCATTTTTCTTGGTGGA
MW429551                            AATCATGGGAAGAATAAGACTACAGTACATGGTTACTTCTCTTCCATTTTTCTTGGTGGA
                                    ************************************************************

AB212830.1                          GTGGATAGTCTAGTTGACTTGATGAACAAGAGCTTTCCTGAGTTGGGTATTAAAAAAACT
NC_044378.1:25821957-25823591       GTGGATAGTCTAGTTGACTTGATGAACAAGAGCTTTCCTGAGTTGGGTATTAAAAAAACT
QKVJ02004887.1:13943-15577          GTGGATAGTCTAGTTGACTTGATGAACAAGAGCTTTCCTGAGTTGGGTATTAAAAAAACT
MNPR01002882.1:12890-14524          GTGGATAGTCTAGTTGACTTGATGAACAAGAGCTTTCCTGAGTTGGGTATTAAAAAAACT
MXBD01006695.1:30197-31831          GTGGATAGTCTAGTTGACTTGATGAACAAGAGCTTTCCTGAGTTGGGTATTAAAAAAACT
AGQN03005496.1:2986-4620            GTGGATAGTCTAGTTGACTTGATGAACAAGAGCTTTCCTGAGTTGGGTATTAAAAAAACT
QVPT02000161.1:1531540-1533174      GTGGATAGTCTAGTTGACTTGATGAACAAGAGCTTTCCTGAGTTGGGTATTAAAAAAACT
MW429551                            GTGGATAGTCTAGTTGACTTGATGAACAAGAGCTTTCCTGAGTTGGGTATTAAAAAAACT
                                    ************************************************************

AB212830.1                          GATTGCAAAGAATTGAGCTGGATTGATACAACCATCTTCTACAGTGGTGTTGTAAATTAC
NC_044378.1:25821957-25823591       GATTGCAAAGAATTGAGCTGGATTGATACAACCATCTTCTACAGTGGTGTTGTAAATTAC
QKVJ02004887.1:13943-15577          GATTGCAAAGAATTGAGCTGGATTGATACAACCATCTTCTACAGTGGTGTTGTAAATTAC
MNPR01002882.1:12890-14524          GATTGCAAAGAATTGAGCTGGATTGATACAACCATCTTCTACAGTGGTGTTGTAAATTAC
MXBD01006695.1:30197-31831          GATTGCAAAGAATTGAGCTGGATTGATACAACCATCTTCTACAGTGGTGTTGTAAATTAC
AGQN03005496.1:2986-4620            GATTGCAAAGAATTGAGCTGGATTGATACAACCATCTTCTACAGTGGTGTTGTAAATTAC
QVPT02000161.1:1531540-1533174      GATTGCAAAGAATTGAGCTGGATTGATACAACCATCTTCTACAGTGGTGTTGTAAATTAC
MW429551                            GATTGCAAAGAATTGAGCTGGATTGATACAACCATCTTCTACAGTGGTGTTGTAAATTAC
                                    ************************************************************

AB212830.1                          AACACTGCTAATTTTAAAAAGGAAATTTTGCTTGATAGATCAGCTGGGAAGAAGACGGCT
NC_044378.1:25821957-25823591       AACACTGCTAATTTTAAAAAGGAAATTTTGCTTGATAGATCAGCTGGGAAGAAGACGGCT
QKVJ02004887.1:13943-15577          AACACTGCTAATTTTAAAAAGGAAATTTTGCTTGATAGATCAGCTGGGAAGAAGACGGCT
MNPR01002882.1:12890-14524          AACACTGCTAATTTTAAAAAGGAAATTTTGCTTGATAGATCAGCTGGGAAGAAGACGGCT
MXBD01006695.1:30197-31831          AACACTGCTAATTTTAAAAAGGAAATTTTGCTTGATAGATCAGCTGGGAAGAAGACGGCT
AGQN03005496.1:2986-4620            AACACTGCTAATTTTAAAAAGGAAATTTTGCTTGATAGATCAGCTGGGAAGAAGACGGCT
QVPT02000161.1:1531540-1533174      AACACTGCTAATTTTAAAAAGGAAATTTTGCTTGATAGATCAGCTGGGAAGAAGACGGCT
MW429551                            AACACTGCTAATTTTAAAAAGGAAATTTTGCTTGATAGATCAGCTGGGAAGAAGACGGCT
                                    ************************************************************

AB212830.1                          TTCTCAATTAAGTTAGACTATGTTAAGAAACTAATACCTGAAACTGCAATGGTCAAAATT
NC_044378.1:25821957-25823591       TTCTCAATTAAGTTAGACTATGTTAAGAAACTAATACCTGAAACTGCAATGGTCAAAATT
QKVJ02004887.1:13943-15577          TTCTCAATTAAGTTAGACTATGTTAAGAAACTAATACCTGAAACTGCAATGGTCAAAATT
MNPR01002882.1:12890-14524          TTCTCAATTAAGTTAGACTATGTTAAGAAACTAATACCTGAAACTGCAATGGTCAAAATT
MXBD01006695.1:30197-31831          TTCTCAATTAAGTTAGACTATGTTAAGAAACTAATACCTGAAACTGCAATGGTCAAAATT
AGQN03005496.1:2986-4620            TTCTCAATTAAGTTAGACTATGTTAAGAAACTAATACCTGAAACTGCAATGGTCAAAATT
QVPT02000161.1:1531540-1533174      TTCTCAATTAAGTTAGACTATGTTAAGAAACTAATACCTGAAACTGCAATGGTCAAAATT
MW429551                            TTCTCAATTAAGTTAGACTATGTTAAGAAACTAATACCTGAAACTGCAATGGTCAAAATT
                                    ************************************************************

AB212830.1                          TTGGAAAAATTATATGAAGAAGAGGTAGGAGTTGGGATGTATGTGTTGTACCCTTACGGT
NC_044378.1:25821957-25823591       TTGGAAAAATTATATGAAGAAGAGGTAGGAGTTGGGATGTATGTGTTGTACCCTTACGAT
QKVJ02004887.1:13943-15577          TTGGAAAAATTATATGAAGAAGAGGTAGGAGTTGGGATGTATGTGTTGTACCCTTACGAT
MNPR01002882.1:12890-14524          TTGGAAAAATTATATGAAGAAGAGGTAGGAGTTGGGATGTATGTGTTGTACCCTTACGGT
MXBD01006695.1:30197-31831          TTGGAAAAATTATATGAAGAAGAGGTAGGAGTTGGGATGTATGTGTTGTACCCTTACGGT
AGQN03005496.1:2986-4620            TTGGAAAAATTATATGAAGAAGAGGTAGGAGTTGGGATGTATGTGTTGTACCCTTACGGT
QVPT02000161.1:1531540-1533174      TTGGAAAAATTATATGAAGAAGAGGTAGGAGTTGGGATGTATGTGTTGTACCCTTACGGT
MW429551                            TTGGAAAAATTATATGAAGAAGAGGTAGGAGTTGGGATGTATGTGTTGTACCCTTACGGT
                                    ********************************************************** *

AB212830.1                          GGTATAATGGATGAGATTTCAGAATCAGCAATTCCATTCCCTCATCGAGCTGGAATAATG
NC_044378.1:25821957-25823591       GGTATAATGGATGAGATTTCAGAATCAGCAATTCCATTCCCTCATCGAGCTGGAATAATG
QKVJ02004887.1:13943-15577          GGTATAATGGATGAGATTTCAGAATCAGCAATTCCATTCCCTCATCGAGCTGGAATAATG
MNPR01002882.1:12890-14524          GGTATAATGGATGAGATTTCAGAATCAGCAATTCCATTCCCTCATCGAGCTGGAATAATG
MXBD01006695.1:30197-31831          GGTATAATGGATGAGATTTCAGAATCAGCAATTCCATTCCCTCATCGAGCTGGAATAATG
AGQN03005496.1:2986-4620            GGTATAATGGATGAGATTTCAGAATCAGCAATTCCATTCCCTCATCGAGCTGGAATAATG
QVPT02000161.1:1531540-1533174      GGTATAATGGATGAGATTTCAGAATCAGCAATTCCATTCCCTCATCGAGCTGGAATAATG
MW429551                            GGTATAATGGATGAGATTTCAGAATCAGCAATTCCATTCCCTCATCGAGCTGGAATAATG
                                    ************************************************************

AB212830.1                          TATGAACTTTGGTACACTGCTACCTGGGAGAAGCAAGAAGATAACGAAAAGCATATAAAC
NC_044378.1:25821957-25823591       TATGAACTTTGGTACACTGCTACCTGGGAGAAGCAAGAAGATAACGAAAAGCATATAAAC
QKVJ02004887.1:13943-15577          TATGAACTTTGGTACACTGCTACCTGGGAGAAGCAAGAAGATAACGAAAAGCATATAAAC
MNPR01002882.1:12890-14524          TATGAACTTTGGTACACTGCTACCTGGGAGAAGCAAGAAGATAACGAAAAGCATATAAAC
MXBD01006695.1:30197-31831          TATGAACTTTGGTACACTGCTACCTGGGAGAAGCAAGAAGATAACGAAAAGCATATAAAC
AGQN03005496.1:2986-4620            TATGAACTTTGGTACACTGCTACCTGGGAGAAGCAAGAAGATAACGAAAAGCATATAAAC
QVPT02000161.1:1531540-1533174      TATGAACTTTGGTACACTGCTACCTGGGAGAAGCAAGAAGATAACGAAAAGCATATAAAC
```

```
MW429551                               TATGAACTTTGGTACACTGCTACCTGGGAGAAGCAAGAAGATAACGAAAAGCATATAAAC
                                       ************************************************************

AB212830.1                             TGGGTTCGAAGTGTTTATAATTTCACAACTCCTTATGTGTCCCAAAATCCAAGATTGGCG
NC_044378.1:25821957-25823591          TGGGTTCGAAGTGTTTATAATTTCACAACTCCTTGTGTGTCCCAAAATCCAAGATTGGCG
QKVJ02004887.1:13943-15577             TGGGTTCGAAGTGTTTATAATTTCACAACTCCTTGTGTGTCCCAAAATCCAAGATTGGCG
MNPR01002882.1:12890-14524             TGGGTTCGAAGTGTTTATAATTTCACAACTCCTTATGTGTCCCAAAATCCAAGATTGGCG
MXBD01006695.1:30197-31831             TGGGTTCGAAGTGTTTATAATTTCACAACTCCTTATGTGTCCCAAAATCCAAGATTGGCG
AGQN03005496.1:2986-4620               TGGGTTCGAAGTGTTTATAATTTCACAACTCCTTATGTGTCCCAAAATCCAAGATTGGCG
QVPT02000161.1:1531540-1533174         TGGGTTCGAAGTGTTTATAATTTCACAACTCCTTATGTGTCCCAAAATCCAAGATTGGCG
MW429551                               TGGGTTCGAAGTGTTTATAATTTCACAACTCCTTATGTGTCCCAAAATCCAAGATTGGCG
                                       ********************************** *************************

AB212830.1                             TATCTCAATTATAGGGACCTTGATTTAGGAAAAACTAATCCTGAGAGTCCTAATAATTAC
NC_044378.1:25821957-25823591          TATCTCAATTATAGGGACCTTGATTTAGGAAAATCTAATCCTGAGAGTCCTAATAATTAC
QKVJ02004887.1:13943-15577             TATCTCAATTATAGGGACCTTGATTTAGGAAAATCTAATCCTGAGAGTCCTAATAATTAC
MNPR01002882.1:12890-14524             TATCTCAATTATAGGGACCTTGATTTAGGAAAAACTAATCCTGAGAGTCCTAATAATTAC
MXBD01006695.1:30197-31831             TATCTCAATTATAGGGACCTTGATTTAGGAAAAACTAATCCTGAGAGTCCTAATAATTAC
AGQN03005496.1:2986-4620               TATCTCAATTATAGGGACCTTGATTTAGGAAAAACTAATCCTGAGAGTCCTAATAATTAC
QVPT02000161.1:1531540-1533174         TATCTCAATTATAGGGACCTTGATTTAGGAAAAACTAATCCTGAGAGTCCTAATAATTAC
MW429551                               TATCTCAATTATAGGGACCTTGATTTAGGAAAAACTAATCCTGAGAGTCCTAATAATTAC
                                       ********************************* **************************

AB212830.1                             ACACAAGCACGTATTTGGGGTGAAAAGTATTTTGGTAAAAATTTTAACAGGTTAGTTAAG
NC_044378.1:25821957-25823591          ACACAAGCACGTATTTGGGGTGAAAAGTATTTTGGTAAAAATTTTAACAGGTTAGTTAAG
QKVJ02004887.1:13943-15577             ACACAAGCACGTATTTGGGGTGAAAAGTATTTTGGTAAAAATTTTAACAGGTTAGTTAAG
MNPR01002882.1:12890-14524             ACACAAGCACGTATTTGGGGTGAAAAGTATTTTGGTAAAAATTTTAACAGGTTAGTTAAG
MXBD01006695.1:30197-31831             ACACAAGCACGTATTTGGGGTGAAAAGTATTTTGGTAAAAATTTTAACAGGTTAGTTAAG
AGQN03005496.1:2986-4620               ACACAAGCACGTATTTGGGGTGAAAAGTATTTTGGTAAAAATTTTAACAGGTTAGTTAAG
QVPT02000161.1:1531540-1533174         ACACAAGCACGTATTTGGGGTGAAAAGTATTTTGGTAAAAATTTTAACAGGTTAGTTAAG
MW429551                               ACACAAGCACGTATTTGGGGTGAAAAGTATTTTGGTAAAAATTTTAACAGGTTAGTTAAG
                                       ************************************************************

AB212830.1                             GTGAAAACCAAAGCTGATCCCAATAATTTTTTTAGAAACGAACAAAGTATCCCACCTCTT
NC_044378.1:25821957-25823591          GTGAAAACCAAAGCTGATCCCAATAATTTTTTTAGAAACGAACAAAGTATCCCACCTCTT
QKVJ02004887.1:13943-15577             GTGAAAACCAAAGCTGATCCCAATAATTTTTTTAGAAACGAACAAAGTATCCCACCTCTT
MNPR01002882.1:12890-14524             GTGAAAACCAAAGCTGATCCCAATAATTTTTTTAGAAACGAACAAAGTATCCCACCTCTT
MXBD01006695.1:30197-31831             GTGAAAACCAAAGCTGATCCCAATAATTTTTTTAGAAACGAACAAAGTATCCCACCTCTT
AGQN03005496.1:2986-4620               GTGAAAACCAAAGCTGATCCCAATAATTTTTTTAGAAACGAACAAAGTATCCCACCTCTT
QVPT02000161.1:1531540-1533174         GTGAAAACCAAAGCTGATCCCAATAATTTTTTTAGAAACGAACAAAGTATCCCACCTCTT
MW429551                               GTGAAAACCAAAGCTGATCCCAATAATTTTTTTAGAAACGAACAAAGTATCCCACCTCTT
                                       ************************************************************

AB212830.1                             CCACCGCATCATCAT
NC_044378.1:25821957-25823591          CCACCGCGTCATCAT
QKVJ02004887.1:13943-15577             CCACCGCGTCATCAT
MNPR01002882.1:12890-14524             CCACCGCGTCATCAT
MXBD01006695.1:30197-31831             CCACCGCGTCATCAT
AGQN03005496.1:2986-4620               CCACCGCGTCATCAT
QVPT02000161.1:1531540-1533174         CCACCGCGTCATCAT
MW429551                               CCACCGCGTCATCAT
                                       ******* *******
```

Input sequences:
((5'3') indicates that the downloaded sequences have been converted into their respective inverse complement to perform the alignment)

>MNPR01002882.1:12890-14524 Cannabis sativa cultivar Cannatonic Cannabis.v1_scf2882_q, whole genome shotgun sequence
ATGAATTGCTCAACATTCTCCTTTTGGTTTGTTTGCAAAATAATATTTTTCTTTCTCTCA
TTCAATATCCAAATTTCAATAGCTAATCCTCAAGAAAACTTCCTTAAATGCTTCTCGGAA
TATATTCCTAACAATCCAGCAAATCCAAAATTCATATACACTCAACACGACCAATTGTAT
ATGTCTGTCCTGAATTCGACAATACAAAATCTTAGATTCACCTCTGATACAACCCCAAAA
CCACTCGTTATTGTCACTCCTTCAAATGTCTCCCATATCCAGGCCAGTATTCTCTGCTCC
AAGAAAGTTGGTTTGCAGATTCGAACTCGAAGCGGTGGCCATGATGCTGAGGGTTTGTCC
TACATATCTCAAGTCCCATTTGCTATAGTAGACTTGAGAAACATGCATACGGTCAAAGTA
GATATTCATAGCCAAACTGCGTGGGTTGAAGCCGGAGCTACCCTTGGAGAAGTTTATTAT
TGGATCAATGAGATGAATGAGAATTTTAGTTTTCCTGGTGGGTATTGCCCTACTGTTGGC
GTAGGTGGACACTTTAGTGGAGGAGGCTATGGAGCATTGATGCGAAATTATGGCCTTGCG
GCTGATAATATCATTGATGCACACTTAGTCAATGTTGATGGAAAAGTTCTAGATCGAAAA
TCCATGGGAGAAGATCTATTTTGGGCTATACGTGGTGGAGGAGGAGAAAACTTTGGAATC
ATTGCAGCATGGAAAATCAAACTTGTTGTTGTCCCATCAAAGGCTACTATATTCAGTGTT
AAAAAGAACATGGAGATACATGGGCTTGTCAAGTTATTTAACAAATGGCAAAATATTGCT
TACAAGTATGACAAAGATTTAATGCTCACGACTCACTTCAGAACTAGGAATATTACAGAT
AATCATGGGAAGAATAAGACTACAGTACATGGTTACTTCTCTTCCATTTTTCTTGGTGGA
GTGGATAGTCTAGTTGACTTGATGAACAAGAGCTTTCCTGAGTTGGGTATTAAAAAAACT
GATTGCAAAGAATTGAGCTGGATTGATACAACCATCTTCTACAGTGGTGTTGTAAATTAC
AACACTGCTAATTTTAAAAAGGAAATTTTGCTTGATAGATCAGCTGGGAAGAAGACGGCT
TTCTCAATTAAGTTAGACTATGTTAAGAAACTAATACCTGAAACTGCAATGGTCAAAATT
TTGGAAAAAATTATATGAAGAAGAGGTAGGAGTTGGGATGTATGTGTTGTACCCTTACGGT
GGTATAATGGATGAGATTTCAGAATCAGCAATTCCATTCCCTCATCGAGCTGGAATAATG
TATGAACTTTGGTACACTGCTACCTGGGAGAAGCAAGAAGATAACGAAAAGCATATAAAC
TGGGTTCGAAGTGTTTATAATTTCACAACTCCTTATGTGTCCCAAAATCCAAGATTGGCG
TATCTCAATTATAGGGACCTTGATTTAGGAAAAACTAATCCTGAGAGTCCTAATAATTAC
ACACAAGCACGTATTTGGGGTGAAAAGTATTTTGGTAAAAATTTTAACAGGTTAGTTAAG
GTGAAAACCAAAGCTGATCCCAATAATTTTTTTAGAAACGAACAAAGTATCCCACCTCTT
CCACCGCGTCATCAT
>MXBD01006695.1:30197-31831 Cannabis sativa cultivar Pineapple Banana Bubba Kush 006705Cannabis_1597M_SHL, whole genome shotgun sequence (5'3')
ATGAATTGCTCAACATTCTCCTTTTGGTTTGTTTGCAAAATAATATTTTTCTTTCTCTCA
TTCAATATCCAAATTTCAATAGCTAATCCTCAAGAAAACTTCCTTAAATGCTTCTCGGAA
TATATTCCTAACAATCCAGCAAATCCAAAATTCATATACACTCAACACGACCAATTGTAT
ATGTCTGTCCTGAATTCGACAATACAAAATCTTAGATTCACCTCTGATACAACCCCAAAA
CCACTCGTTATTGTCACTCCTTCAAATGTCTCCCATATCCAGGCCAGTATTCTCTGCTCC
AAGAAAGTTGGTTTGCAGATTCGAACTCGAAGCGGTGGCCATGATGCTGAGGGTTTGTCC
TACATATCTCAAGTCCCATTTGCTATAGTAGACTTGAGAAACATGCATACGGTCAAAGTA
GATATTCATAGCCAAACTGCGTGGGTTGAAGCCGGAGCTACCCTTGGAGAAGTTTATTAT
TGGATCAATGAGATGAATGAGAATTTTAGTTTTCCTGGTGGGTATTGCCCTACTGTTGGC
GTAGGTGGACACTTTAGTGGAGGAGGCTATGGAGCATTGATGCGAAATTATGGCCTTGCG
GCTGATAATATCATTGATGCACACTTAGTCAATGTTGATGGAAAAGTTCTAGATCGAAAA
TCCATGGGAGAAGATCTATTTTGGGCTATACGTGGTGGAGGAGGAGAAAACTTTGGAATC
ATTGCAGCATGGAAAATCAAACTTGTTGTTGTCCCATCAAAGGCTACTATATTCAGTGTT
AAAAAGAACATGGAGATACATGGGCTTGTCAAGTTATTTAACAAATGGCAAAATATTGCT
TACAAGTATGACAAAGATTTAATGCTCACGACTCACTTCAGAACTAGGAATATTACAGAT
AATCATGGGAAGAATAAGACTACAGTACATGGTTACTTCTCTTCCATTTTTCTTGGTGGA
GTGGATAGTCTAGTTGACTTGATGAACAAGAGCTTTCCTGAGTTGGGTATTAAAAAAACT
GATTGCAAAGAATTGAGCTGGATTGATACAACCATCTTCTACAGTGGTGTTGTAAATTAC
AACACTGCTAATTTTAAAAAGGAAATTTTGCTTGATAGATCAGCTGGGAAGAAGACGGCT
TTCTCAATTAAGTTAGACTATGTTAAGAAACTAATACCTGAAACTGCAATGGTCAAAATT
TTGGAAAAAATTATATGAAGAAGAGGTAGGAGTTGGGATGTATGTGTTGTACCCTTACGGT
GGTATAATGGATGAGATTTCAGAATCAGCAATTCCATTCCCTCATCGAGCTGGAATAATG
TATGAACTTTGGTACACTGCTACCTGGGAGAAGCAAGAAGATAACGAAAAGCATATAAAC
TGGGTTCGAAGTGTTTATAATTTCACAACTCCTTATGTGTCCCAAAATCCAAGATTGGCG
TATCTCAATTATAGGGACCTTGATTTAGGAAAAACTAATCCTGAGAGTCCTAATAATTAC
ACACAAGCACGTATTTGGGGTGAAAAGTATTTTGGTAAAAATTTTAACAGGTTAGTTAAG

GTGAAAACCAAAGCTGATCCCAATAATTTTTTTAGAAACGAACAAAGTATCCCACCTCTT
CCACCGCGTCATCAT
>AGQN03005496.1:2986-4620 Cannabis sativa cultivar Purple Kush 005500F, whole genome shotgun sequence (5'3')
ATGAATTGCTCAACATTCTCCTTTTGGTTTGTTTGCAAAATAATATTTTTCTTTCTCTCA
TTCAATATCCAAATTTCAATAGCTAATCCTCAAGAAAACTTCCTTAAATGCTTCTCGGAA
TATATTCCTAACAATCCAGCAAATCCAAAATTCATATACACTCAACACGACCAATTGTAT
ATGTCTGTCCTGAATTCGACAATACAAAATCTTAGATTCACCTCTGATACAACCCCAAAA
CCACTCGTTATTGTCACTCCTTCAAATGTCTCCCATATCCAGGCCAGTATTCTCTGCTCC
AAGAAAGTTGGTTTGCAGATTCGAACTCGAAGCGGTGGCCATGATGCTGAGGGTTTGTCC
TACATATCTCAAGTCCCATTTGCTATAGTAGACTTGAGAAACATGCATACGGTCAAAGTA
GATATTCATAGCCAAACTGCGTGGGTTGAAGCCGGAGCTACCCTTGGAGAAGTTTATTAT
TGGATCAATGAGATGAATGAGAATTTTAGTTTTCCTGGTGGGTATTGCCCTACTGTTGGC
GTAGGTGGACACTTTAGTGGAGGAGGCTATGGAGCATTGATGCGAAATTATGGCCTTGCG
GCTGATAATATCATTGATGCACACTTAGTCAATGTTGATGGAAAAGTTCTAGATCGAAAA
TCCATGGGAGAAGATCTATTTTGGGCTATACGTGGTGGAGGAGGAGAAAACTTTGGAATC
ATTGCAGCATGGAAAATCAAACTTGTTGTTGTCCCATCAAAGGCTACTATATTCAGTGTT
AAAAAGAACATGGAGATACATGGGCTTGTCAAGTTATTTAACAAATGGCAAAATATTGCT
TACAAGTATGACAAAGATTTAATGCTCACGACTCACTTCAGAACTAGGAATATTACAGAT
AATCATGGGAAGAATAAGACTACAGTACATGGTTACTTCTCTTCCATTTTTCTTGGTGGA
GTGGATAGTCTAGTTGACTTGATGAACAAGAGCTTTCCTGAGTTGGGTATTAAAAAAACT
GATTGCAAAGAATTGAGCTGGATTGATACAACCATCTTCTACAGTGGTGTTGTAAATTAC
AACACTGCTAATTTTAAAAAGGAAATTTTGCTTGATAGATCAGCTGGGAAGAAGACGGCT
TTCTCAATTAAGTTAGACTATGTTAAGAAACTAATACCTGAAACTGCAATGGTCAAAATT
TTGGAAAAATTATATGAAGAAGAGGTAGGAGTTGGGATGTATGTGTTGTACCCTTACGGT
GGTATAATGGATGAGATTTCAGAATCAGCAATTCCATTCCCTCATCGAGCTGGAATAATG
TATGAACTTTGGTACACTGCTACCTGGGAGAAGCAAGAAGATAACGAAAAGCATATAAAC
TGGGTTCGAAGTGTTTATAATTTCACAACTCCTTATGTGTCCCAAAATCCAAGATTGGCG
TATCTCAATTATAGGGACCTTGATTTAGGAAAAACTAATCCTGAGAGTCCTAATAATTAC
ACACAAGCACGTATTTGGGGTGAAAAGTATTTTGGTAAAAATTTTAACAGGTTAGTTAAG
GTGAAAACCAAAGCTGATCCCAATAATTTTTTTAGAAACGAACAAAGTATCCCACCTCTT
CCACCGCGTCATCAT
>QVPT02000161.1:1531540-1533174 Cannabis sativa cultivar Jamaican Lion DASH 000163F_arrow, whole genome shotgun sequence (5'3')
ATGAATTGCTCAACATTCTCCTTTTGGTTTGTTTGCAAAATAATATTTTTCTTTCTCTCA
TTCAATATCCAAATTTCAATAGCTAATCCTCAAGAAAACTTCCTTAAATGCTTCTCGGAA
TATATTCCTAACAATCCAGCAAATCCAAAATTCATATACACTCAACACGACCAATTGTAT
ATGTCTGTCCTGAATTCGACAATACAAAATCTTAGATTCACCTCTGATACAACCCCAAAA
CCACTCGTTATTGTCACTCCTTCAAATGTCTCCCATATCCAGGCCAGTATTCTCTGCTCC
AAGAAAGTTGGTTTGCAGATTCGAACTCGAAGCGGTGGCCATGATGCTGAGGGTTTGTCC
TACATATCTCAAGTCCCATTTGCTATAGTAGACTTGAGAAACATGCATACGGTCAAAGTA
GATATTCATAGCCAAACTGCGTGGGTTGAAGCCGGAGCTACCCTTGGAGAAGTTTATTAT
TGGATCAATGAGATGAATGAGAATTTTAGTTTTCCTGGTGGGTATTGCCCTACTGTTGGC
GTAGGTGGACACTTTAGTGGAGGAGGCTATGGAGCATTGATGCGAAATTATGGCCTTGCG
GCTGATAATATCATTGATGCACACTTAGTCAATGTTGATGGAAAAGTTCTAGATCGAAAA
TCCATGGGAGAAGATCTATTTTGGGCTATACGTGGTGGAGGAGGAGAAAACTTTGGAATC
ATTGCAGCATGGAAAATCAAACTTGTTGTTGTCCCATCAAAGGCTACTATATTCAGTGTT
AAAAAGAACATGGAGATACATGGGCTTGTCAAGTTATTTAACAAATGGCAAAATATTGCT
TACAAGTATGACAAAGATTTAATGCTCACGACTCACTTCAGAACTAGGAATATTACAGAT
AATCATGGGAAGAATAAGACTACAGTACATGGTTACTTCTCTTCCATTTTTCTTGGTGGA
GTGGATAGTCTAGTTGACTTGATGAACAAGAGCTTTCCTGAGTTGGGTATTAAAAAAACT
GATTGCAAAGAATTGAGCTGGATTGATACAACCATCTTCTACAGTGGTGTTGTAAATTAC
AACACTGCTAATTTTAAAAAGGAAATTTTGCTTGATAGATCAGCTGGGAAGAAGACGGCT
TTCTCAATTAAGTTAGACTATGTTAAGAAACTAATACCTGAAACTGCAATGGTCAAAATT
TTGGAAAAATTATATGAAGAAGAGGTAGGAGTTGGGATGTATGTGTTGTACCCTTACGGT
GGTATAATGGATGAGATTTCAGAATCAGCAATTCCATTCCCTCATCGAGCTGGAATAATG
TATGAACTTTGGTACACTGCTACCTGGGAGAAGCAAGAAGATAACGAAAAGCATATAAAC
TGGGTTCGAAGTGTTTATAATTTCACAACTCCTTATGTGTCCCAAAATCCAAGATTGGCG
TATCTCAATTATAGGGACCTTGATTTAGGAAAAACTAATCCTGAGAGTCCTAATAATTAC
ACACAAGCACGTATTTGGGGTGAAAAGTATTTTGGTAAAAATTTTAACAGGTTAGTTAAG
GTGAAAACCAAAGCTGATCCCAATAATTTTTTTAGAAACGAACAAAGTATCCCACCTCTT
CCACCGCGTCATCAT
>NC_044378.1:25821957-25823591 Cannabis sativa chromosome 7, cs10

ATGAATTGCTCAACATTCTCCTTTTGGTTTGTTTGCAAAATAATATTTTTCTTTCTCTCA
TTCAATATCCAAATTTCAATAGCTAATCCTCAAGAAAACTTCCTTAAATGCTTCTCGGAA
TATATTCCTAACAATCCAGCAAATCCAAAATTCATATACACTCAACACGACCAATTGTAT
ATGTCTGTCCTGAATTCGACAATACAAAATCTTAGATTCACCTCTGATACAACCCCAAAA
CCACTCGTTATTGTCACTCCTTCAAATGTCTCCCATATCCAGGCCAGTATTCTCTGCTCC
AAGAAAGTTGGTTTGCAGATTCGAACTCGAAGCGGTGGCCATGATGCTGAGGGTTTGTCC
TACATATCTCAAGTCCCATTTGCTATAGTAGACTTGAGAAACATGCATACGGTCAAAGTA
GATATTCATAGCCAAACTGCGTGGGTTGAAGCCGGAGCTACCCTTGGAGAAGTTTATTAT
TGGATCAATGAGATGAATGAGAATTTTAGTTTTCCTGGTGGGTATTGCCCTACTGTTGGC
GTAGGTGGACACTTTAGTGGAGGAGGCTATGGAGCATTGATGCGAAATTATGGCCTTGCG
GCTGATAATATCATTGATGCACACTTAGTCAATGTTGATGGAAAAGTTCTAGATCGAAAA
TCCATGGGAGAAGATCTATTTTGGGCTATACGTGGTGGAGGAGGAGAAAACTTTGGAATC
ATTGCAGCATGGAAAATCAAACTTGTTGTTGTCCCATCAAAGGCTACTATATTCAGTGTT
AAAAAGAACATGGAGATACATGGGCTTGTCAAGTTATTTAACAAATGGCAAAATATTGCT
TACAAGTATGACAAAGATTTAATGCTCACGACTCACTTCAGAACTAGGAATATTACAGAT
AATCATGGGAAGAATAAGACTACAGTACATGGTTACTTCTCTTCCATTTTTCTTGGTGGA
GTGGATAGTCTAGTTGACTTGATGAACAAGAGCTTTCCTGAGTTGGGTATTAAAAAAACT
GATTGCAAAGAATTGAGCTGGATTGATACAACCATCTTCTACAGTGGTGTTGTAAATTAC
AACACTGCTAATTTTAAAAAGGAAATTTTGCTTGATAGATCAGCTGGGAAGAAGACGGCT
TTCTCAATTAAGTTAGACTATGTTAAGAAACTAATACCTGAAACTGCAATGGTCAAAATT
TTGGAAAAATTATATGAAGAAGAGGTAGGAGTTGGGATGTATGTGTTGTACCCTTACGAT
GGTATAATGGATGAGATTTCAGAATCAGCAATTCCATTCCCTCATCGAGCTGGAATAATG
TATGAACTTTGGTACACTGCTACCTGGGAGAAGCAAGAAGATAACGAAAAGCATATAAAC
TGGGTTCGAAGTGTTTATAATTTCACAACTCCTTGTGTGTCCCAAAATCCAAGATTGGCG
TATCTCAATTATAGGGACCTTGATTTAGGAAAATCTAATCCTGAGAGTCCTAATAATTAC
ACACAAGCACGTATTTGGGGTGAAAAGTATTTTGGTAAAAATTTTAACAGGTTAGTTAAG
GTGAAAACCAAAGCTGATCCCAATAATTTTTTTAGAAACGAACAAAGTATCCCACCTCTT
CCACCGCGTCATCAT
>QKVJ02004887.1:13943-15577 Cannabis sativa cultivar Finola 004887F, whole genome shotgun sequence (5'3')
ATGAATTGCTCAACATTCTCCTTTTGGTTTGTTTGCAAAATAATATTTTTCTTTCTCTCA
TTCAATATCCAAATTTCAATAGCTAATCCTCAAGAAAACTTCCTTAAATGCTTCTCGGAA
TATATTCCTAACAATCCAGCAAATCCAAAATTCATATACACTCAACACGACCAATTGTAT
ATGTCTGTCCTGAATTCGACAATACAAAATCTTAGATTCACCTCTGATACAACCCCAAAA
CCACTCGTTATTGTCACTCCTTCAAATGTCTCCCATATCCAGGCCAGTATTCTCTGCTCC
AAGAAAGTTGGTTTGCAGATTCGAACTCGAAGCGGTGGCCATGATGCTGAGGGTTTGTCC
TACATATCTCAAGTCCCATTTGCTATAGTAGACTTGAGAAACATGCATACGGTCAAAGTA
GATATTCATAGCCAAACTGCGTGGGTTGAAGCCGGAGCTACCCTTGGAGAAGTTTATTAT
TGGATCAATGAGATGAATGAGAATTTTAGTTTTCCTGGTGGGTATTGCCCTACTGTTGGC
GTAGGTGGACACTTTAGTGGAGGAGGCTATGGAGCATTGATGCGAAATTATGGCCTTGCG
GCTGATAATATCATTGATGCACACTTAGTCAATGTTGATGGAAAAGTTCTAGATCGAAAA
TCCATGGGAGAAGATCTATTTTGGGCTATACGTGGTGGAGGAGGAGAAAACTTTGGAATC
ATTGCAGCATGGAAAATCAAACTTGTTGTTGTCCCATCAAAGGCTACTATATTCAGTGTT
AAAAAGAACATGGAGATACATGGGCTTGTCAAGTTATTTAACAAATGGCAAAATATTGCT
TACAAGTATGACAAAGATTTAATGCTCACGACTCACTTCAGAACTAGGAATATTACAGAT
AATCATGGGAAGAATAAGACTACAGTACATGGTTACTTCTCTTCCATTTTTCTTGGTGGA
GTGGATAGTCTAGTTGACTTGATGAACAAGAGCTTTCCTGAGTTGGGTATTAAAAAAACT
GATTGCAAAGAATTGAGCTGGATTGATACAACCATCTTCTACAGTGGTGTTGTAAATTAC
AACACTGCTAATTTTAAAAAGGAAATTTTGCTTGATAGATCAGCTGGGAAGAAGACGGCT
TTCTCAATTAAGTTAGACTATGTTAAGAAACTAATACCTGAAACTGCAATGGTCAAAATT
TTGGAAAAATTATATGAAGAAGAGGTAGGAGTTGGGATGTATGTGTTGTACCCTTACGAT
GGTATAATGGATGAGATTTCAGAATCAGCAATTCCATTCCCTCATCGAGCTGGAATAATG
TATGAACTTTGGTACACTGCTACCTGGGAGAAGCAAGAAGATAACGAAAAGCATATAAAC
TGGGTTCGAAGTGTTTATAATTTCACAACTCCTTGTGTGTCCCAAAATCCAAGATTGGCG
TATCTCAATTATAGGGACCTTGATTTAGGAAAATCTAATCCTGAGAGTCCTAATAATTAC
ACACAAGCACGTATTTGGGGTGAAAAGTATTTTGGTAAAAATTTTAACAGGTTAGTTAAG
GTGAAAACCAAAGCTGATCCCAATAATTTTTTTAGAAACGAACAAAGTATCCCACCTCTT
CCACCGCGTCATCAT
>AB212830.1 Cannabis sativa gene for tetrahydrocannabinolic acid synthase, partial cds
ATGAATTGCTCAGCATTTTCCTTTTGGTTTGTTTGCAAAATAATATTTTTCTTTCTCTCA
TTCAATATCCAAATTTCAATAGCTAATCCTCAAGAAAACTTCCTTAAATGCTTCTCGGAA
TATATTCCTAACAATCCAGCAAATCCAAAATTCATATACACTCAACACGACCAATTGTAT

ATGTCTGTCCTGAATTCGACAATACAAAATCTTAGATTCACCTCTGATACAACCCCAAAA
CCACTCGTTATTGTCACTCCTTCAAATGTCTCCCATATCCAGGCCAGTATTCTCTGCTCC
AAGAAAGTTGGTTTGCAGATTCGAACTCGAAGCGGTGGCCATGATGCTGAGGGTTTGTCC
TACATATCTCAAGTCCCATTTGCTATAGTAGACTTGAGAAACATGCATACGGTCAAAGTA
GATATTCATAGCCAAACTGCGTGGGTTGAAGCCGGAGCTACCCTTGGAGAAGTTTATTAT
TGGATCAATGAGATGAATGAGAATTTTAGTTTTCCTGGTGGGTATTGCCCTACTGTTGGC
GTAGGTGGACACTTTAGTGGAGGAGGCTATGGAGCATTGATGCGAAATTATGGCCTTGCG
GCTGATAATATCATTGATGCACACTTAGTCAATGTTGATGGAAAAGTTCTAGATCGAAAA
TCCATGGGAGAAGATCTATTTTGGGCTATACGTGGTGGAGGAGGAGAAAACTTTGGAATC
ATTGCAGCATGGAAAATCAAACTTGTTGTTGTCCCATCAAAGGCTACTATATTCAGTGTT
AAAAAGAACATGGAGATACATGGGCTTGTCAAGTTATTTAACAAATGGCAAAATATTGCT
TACAAGTATGACAAAGATTTAATGCTCACGACTCACTTCAGAACTAGGAATATTACAGAT
AATCATGGGAAGAATAAGACTACAGTACATGGTTACTTCTCTTCCATTTTTCTTGGTGGA
GTGGATAGTCTAGTTGACTTGATGAACAAGAGCTTTCCTGAGTTGGGTATTAAAAAAACT
GATTGCAAAGAATTGAGCTGGATTGATACAACCATCTTCTACAGTGGTGTTGTAAATTAC
AACACTGCTAATTTTAAAAAGGAAATTTTGCTTGATAGATCAGCTGGGAAGAAGACGGCT
TTCTCAATTAAGTTAGACTATGTTAAGAAACTAATACCTGAAACTGCAATGGTCAAAATT
TTGGAAAAATTATATGAAGAAGAGGTAGGAGTTGGGATGTATGTGTTGTACCCTTACGGT
GGTATAATGGATGAGATTTCAGAATCAGCAATTCCATTCCCTCATCGAGCTGGAATAATG
TATGAACTTTGGTACACTGCTACCTGGGAGAAGCAAGAAGATAACGAAAAGCATATAAAC
TGGGTTCGAAGTGTTTATAATTTCACAACTCCTTATGTGTCCCAAAATCCAAGATTGGCG
TATCTCAATTATAGGGACCTTGATTTAGGAAAAACTAATCCTGAGAGTCCTAATAATTAC
ACACAAGCACGTATTTGGGGTGAAAAGTATTTTGGTAAAAATTTTAACAGGTTAGTTAAG
GTGAAAACCAAAGCTGATCCCAATAATTTTTTTAGAAACGAACAAAGTATCCCACCTCTT
CCACCGCATCATCAT
>MW429551
ATGAATTGCTCAACATTCTCCTTTTGGTTTGTTTGCAAAATAATATTTTTCTTTCTCTCA
TTCAATATCCAAATTTCAATAGCTAATCCTCAAGAAAACTTCCTTAAATGCTTCTCGGAA
TATATTCCTAACAATCCAGCAAATCCAAAATTCATATACACTCAACACGACCAATTGTAT
ATGTCTGTCCTGAATTCGACAATACAAAATCTTAGATTCACCTCTGATACAACCCCAAAA
CCACTCGTTATTGTCACTCCTTCAAATGTCTCCCATATCCAGGCCAGTATTCTCTGCTCC
AAGAAAGTTGGTTTGCAGATTCGAACTCGAAGCGGTGGCCATGATGCTGAGGGTTTGTCC
TACATATCTCAAGTCCCATTTGCTATAGTAGACTTGAGAAACATGCATACGGTCAAAGTA
GATATTCATAGCCAAACTGCGTGGGTTGAAGCCGGAGCTACCCTTGGAGAAGTTTATTAT
TGGATCAATGAGATGAATGAGAATTTTAGTTTTCCTGGTGGGTATTGCCCTACTGTTGGC
GTAGGTGGACACTTTAGTGGAGGAGGCTATGGAGCATTGATGCGAAATTATGGCCTTGCG
GCTGATAATATCATTGATGCACACTTAGTCAATGTTGATGGAAAAGTTCTAGATCGAAAA
TCCATGGGAGAAGATCTATTTTGGGCTATACGTGGTGGAGGAGGAGAAAACTTTGGAATC
ATTGCAGCATGGAAAATCAAACTTGTTGTTGTCCCATCAAAGGCTACTATATTCAGTGTT
AAAAAGAACATGGAGATACATGGGCTTGTCAAGTTATTTAACAAATGGCAAAATATTGCT
TACAAGTATGACAAAGATTTAATGCTCACGACTCACTTCAGAACTAGGAATATTACAGAT
AATCATGGGAAGAATAAGACTACAGTACATGGTTACTTCTCTTCCATTTTTCTTGGTGGA
GTGGATAGTCTAGTTGACTTGATGAACAAGAGCTTTCCTGAGTTGGGTATTAAAAAAACT
GATTGCAAAGAATTGAGCTGGATTGATACAACCATCTTCTACAGTGGTGTTGTAAATTAC
AACACTGCTAATTTTAAAAAGGAAATTTTGCTTGATAGATCAGCTGGGAAGAAGACGGCT
TTCTCAATTAAGTTAGACTATGTTAAGAAACTAATACCTGAAACTGCAATGGTCAAAATT
TTGGAAAAATTATATGAAGAAGAGGTAGGAGTTGGGATGTATGTGTTGTACCCTTACGGT
GGTATAATGGATGAGATTTCAGAATCAGCAATTCCATTCCCTCATCGAGCTGGAATAATG
TATGAACTTTGGTACACTGCTACCTGGGAGAAGCAAGAAGATAACGAAAAGCATATAAAC
TGGGTTCGAAGTGTTTATAATTTCACAACTCCTTATGTGTCCCAAAATCCAAGATTGGCG
TATCTCAATTATAGGGACCTTGATTTAGGAAAAACTAATCCTGAGAGTCCTAATAATTAC
ACACAAGCACGTATTTGGGGTGAAAAGTATTTTGGTAAAAATTTTAACAGGTTAGTTAAG
GTGAAAACCAAAGCTGATCCCAATAATTTTTTTAGAAACGAACAAAGTATCCCACCTCTT
CCACCGCGTCATCAT
