# Supplementary material for: Analysis of Sequence Variability and Transcriptional Profile of Cannabinoid synthase Genes in Cannabis sativa L. Chemotypes with a Focus on Cannabichromenic acid synthase
Source: Plants (Basel). 2021 Sep 8;10(9):1857. doi: 10.3390/plants10091857 (PMC8466818; doi:10.3390/plants10091857)
Supplement: Supplementary file 1 [file plants-10-01857-s001.zip › Supplementary File 2.pdf]

Supplementary File 2

Analysis of sequence variability and transcriptional profile of *cannabinoid synthase* genes in *Cannabis sativa* L. chemotypes with a focus on *cannabichromenic acid synthase*

Flavia Fulvio[1,2], Roberta Paris[1*], Massimo Montanari[1], Cinzia Citti[3,4], Vincenzo Cilento[1], Laura Bassolino[1], Anna Moschella[1], Ilaria Alberti[5], Nicola Pecchioni[6], Giuseppe Cannazza[3,4] and Giuseppe Mandolino[1]

1 CREA-Research Center for Cereal and Industrial Crops, Via di Corticella 133, 40128 Bologna, Italy

2 Department of Sciences of Agriculture, Food Natural resources and Engineering, University of Foggia, Via Napoli 25, 71122 Foggia, Italy

3 CNR NANOTEC – Institute of Nanotechnology, Via Monteroni, 73100, Lecce, Italy

4 Department of Life Science, University of Modena and Reggio Emilia, Via G. Campi 103, 41125, Modena, Italy

5 CREA-Research Center for Cereal and Industrial Crops, Via G. Amendola 82, 45100 Rovigo, Italy

6 CREA-Research Center for Cereal and Industrial Crops, S.S. 673 Km 25,200, 71122, Foggia, Italy

* Correspondence: roberta.paris@crea.gov.it

SAMPLE

Description	Inflorescences of Cannabis grown outdoor
Microdissection or macrodissection	Macrodissection
Processing procedure	Samples were immediately frozen in liquid nitrogen, then conserved ato -80°C until extraction. Before extraction they were powdered with mortar in liquid nitrogen
Sample storage conditions and duration (especially for FFPE samples)	Up to four weeks at -80°C

NUCLEIC ACID EXTRACTION

Procedure and/or instrumentation	Extraction via commercial kit
Name of kit and details of any modifications	Spectrum™ Plant Total RNA Kit
Details of DNase or RNAse treatment	DNase I
Contamination assessment (DNA or RNA)	No reverse transcription controls
Nucleic acid quantification	Absorbance
Instrument and method	Infinite 200 PRO spectrophotometer (TECAN)
RNA integrity method/instrument	Evaluated on agarose gel stained with gelred

REVERSE TRANSCRIPTION

Complete reaction conditions	RNA samples were added with 2X RT Buffer Mix, 20X RT Enzyme Mix and Nuclease-free H2O up to 20 µl per reaction. Then incubate at 37°C for 60 minutes. The reaction was stopped by heating to 95°C for 5 minutes and hold at 4°C.

Amount of RNA and reaction volume	500 ng total RNA per 20 µl of reaction
Priming oligonucleotide (if using GSP) and concentration	Oligo dT-16 primers
Reverse transcriptase and concentration	MultiScribe™ x (50 U, for 20 µL reactions)
Temperature and time	60 min at 37°C. 5 min at 95°C. hold at 4°C.

qPCR TARGET INFORMATION

Sequence accession number	Shown in Supplementary Tables S3,S4
Amplicon length	Shown in Table 2
In silico specificity screen (BLAST, etc)	Primers were searched by BlastN against Cannabis sativa non-redundant database at NCBI
Location of each primer by exon or intron (if applicable)	Working on single exon genes

qPCR OLIGONUCLEOTIDES

Primer sequences	Shown in Table 2
Location and identity of any modifications	No modifications

qPCR PROTOCOL

Complete reaction conditions	Shown in Appendix B
Reaction volume and amount of cDNA/DNA	Reaction volume: 10 µl, cDNA volume: 1.5 µl
Primer, (probe), Mg++ and dNTP concentrations	Primer:shown in Table 2, for Mg and dNTP concentration refer to PowerUp SYBR Green MasterMix manufacturer's specifications (Thermo Fisher).
Polymerase identity and concentration	Refer to PowerUp SYBR Green MasterMix manufacturer's specifications.
Buffer/kit identity and manufacturer	Refer to PowerUp SYBR Green MasterMix manufacturer's specifications.
Additives (SYBR Green I, DMSO, etc.)	SYBR green
Complete thermocycling parameters	Shown in Appendix B
Manufacturer of qPCR instrument	RotorGene 6000 (Corbett)

qPCR VALIDATION

Specificity (gel, sequence, melt, or digest)	Dissociation curve verificatin
For SYBR Green I, Cq of the NTC	No amplification detected for NTCs
Standard curves with slope and y-intercept	CBDAS slope: -3.289; Intercept: 21.482; THCAS slope: -3.928; Intercept: 24.428; CBCAS slope: -3.289; Intercept: 22.266
PCR efficiency calculated from slope	Shown in Table 3
r2 of standard curve	Shown in Table 3
Linear dynamic range	Samples were situated within the range of the efficiency curve

DATA ANALYSIS

qPCR analysis program (source, version)	Excel
Cq method determination	Automatic threshold determination by RotorGene 6000
Outlier identification and disposition	Dissociation curve verificatin
Results of NTCs	Dissociation curve verificatin
Justification of number and choice of reference genes	Two reference genes were selected, based on evidences illustrated in materials and methods and results.
Description of normalisation method	Raw data for target and reference genes were transformed using the 'Standard Curve Method'; the transcripts level of target genes was normalized to the geometric mean of the transcripts level of CsClathrin and CsRAN and reported as Relative Quantitation (RQ) of transcriptional levels [46] expressed in Arbitrary Units (A.U.). Finally, the standard error of the mean of three biological replicates was calculated and reported in the graphs as bar of error.
Number and stage (RT or qPCR) of technical replicates	Two technical replicates
Statistical methods for result significance	Student't t-test on log transformed values
Software (source, version)	Excel
